# Mapping novel QTLs associated with grain number and primary branching in rice using new plant type derived RILs

**DOI:** 10.1186/s12870-026-08481-2

**Published:** 2026-03-10

**Authors:** Shekharappa Nandakumar, Sonu, Vikram Jeet Singh, Kunnummal Kurungara Vinod, Bheemapura Shivakumar Harshitha, Subbaiyan Gopala Krishnan, Brijesh Kumar Dixit, Shridhar Ragi, Ranjith Kumar Ellur, Haritha Bollinedi, Mariappan Nagarajan, Nanjappa Shivakumar, Tapan Kumar Mondal, Ashok Kumar Singh, Prolay Kumar Bhowmick

**Affiliations:** 1 Division of Genetics, ICAR-Indian Agricultural Research Institute, New Delhi, India; 2 Department of Seed Science and Technology, Acharya Narendra Deva University of Agriculture and Technology, Ayodhya, India; 3 CSB-Institute for Seri-Biotechnological Research, Kodathi, Bengaluru, 560035 India; 4 Rice Breeding and Genetics Research Centre, ICAR-Indian Agricultural Research Institute, Aduthurai, Tamil Nadu India; 5 Zonal Agriculture Research Station, College of Agriculture, University of Agricultural Sciences, Mandya, Bangalore, Karnataka India; 6 ICAR-National Institute of Plant Biotechnology, New Delhi, India; 7 ICAR-Cenral Arid Zone Research Institute, RRS, Jaisalmer, India; 8 Department of Genetics, New Mexico State University, ASC at Clovis, 2346 SR 288, New Mexico, USA

**Keywords:** Rice, QTL mapping, Panicle primary branching, Grain number

## Abstract

**Background:**

The increase in the productivity of rice has become very limited due to limited major genes for key yield-attributing traits, namely, grain number per panicle, thousand-grain weight, panicle primary branching, and productive tiller number. To break this barrier, there’s an immediate demand to identify novel genes or quantitative trait loci (QTLs) from diverse germplasm. This study aims to address this challenge by specifically identifying genes and QTLs controlling grain number and primary branch number in order to facilitate future molecular breeding efforts.

**Results:**

QTL mapping was done using a custom microsatellite linkage map constructed for the Recombinant inbred lines (RILs). RIL population consisting of 175 lines derived from PR126 and Pusa NPT34 were used for QTL mapping, and mapping population was evaluated at three locations namely Delhi, Karnal and Aduthurai. Total of 25 QTLs were identified for nine traits, among which 13 were found distributed in five hotspots. Seven QTLs were having major effects and the remaining showed minor effects on the respective target traits. QTLs, *qFGN9.1* for grain number lying at the marker interval, RM444-RM6920 and *qPBN11.1* for primary branches lying at the marker interval of RM144-RM6965 on chromosome 9 and 11, were found novel respectively. A largest QTL hotspot on chromosome 6, harboured QTLs for GN, PH, PL, PBN, and YLD. Seventeen putative candidate gene models were identified through in silico analysis which involves in inflorescence development. The major gene models include *APETALA* genes, MADS box family proteins and WD40 which directly controls panicle primary branching, spikelet development. Therefore, further fine mapping of marker intervals can help in narrowing the genomic region and trait specific marker development. It enables more precise introgression of QTLs, for primary branches, grain number and other panicle architecture related traits into elite cultivars.

**Conclusion:**

In the present study, QTLs were mapped for grain number, panicle primary branching and yield attributed traits and identified two novel genomic regions for grain number and panicle primary branching. We have identified four QTL hotspots comprising 13 QTLS and the remaining QTLs present outside the hotspots. Further fine mapping of marker intervals can help in narrowing the genomic region and in identifying candidate genes to enable more precise marker-assisted selection for primary branches and grain number.

**Supplementary Information:**

The online version contains supplementary material available at 10.1186/s12870-026-08481-2.

## Background

Rice is cultivated in more than 114 countries spread over six continents [1], where India’s contribution is more than 20% of the world’s rice production [2]. In India, rice production accounts for 47% [3] of the total cereal production and is considered one of the most important dietary sources. Recent estimates suggest that to meet the food demand of the growing world population, which is predicted to increase by 9.7% in 2050, the production of rice must go up by 40% [4]. The current phase of rice cultivation, productivity and production, will not be sufficient enough to feed the future population [2, 5, 6, 7]. The most essential but difficult objective for breeders has been to increase the yield of staple crops [8].

The rice panicle is a raceme with no apical growth and consists of predetermined primary and secondary branches as well as number of spikelets. The genetic mechanism of panicle development is very complex, that begins with the transformation of shoot apical meristem (SAM) and ends with the formation of spiekelets. The branches and their differentiated spikelet meristems will eventually form the basic structure of a rice panicle and determine the spikelet number [9]. The spikelet number is influenced by endogenous factors such as genetic makeup and plant physiology. Therefore, to study the genetics of grain number, it is essential to identify the genomic regions controlling this trait.

Identification of QTLs for yield and yield attributing traits through QTL mapping is an effective strategy to break the yield plateau in rice. With the advancement in the molecular marker technology many QTLs have been identified for grain number, panicle primary branching, panicle length, plant height, tiller number, flowering. Among the several quantitative trait loci (QTLs) which are reported for grain yield namely, *Gn1a*, *NOG1*, *qGN4-1*, *DEP1*, *LAX1*, *IPA1*, *APO1*, in rice are seen to control the grain number primarily [10]. The first QTL identified for grain number in rice was *Gn1a* on chromosome 1, encodes cytokinin degradation enzyme. Reduced expression of *Gn1a* allele increases cytokinin content in the inflorescence meristem thereby increases the grain number per panicle. Similarly, *NUMBER OF GRAINS 1* (*NOG1*) is a gene was, mapped using an *Oryza rufipogon* introgression line SIL176 in a high-yielding *indica* background, Guichao 2. This gene is located on chromosome 1, encodes for an enoyl-CoA hydratase/isomerase (ECH) enzyme, bearing a primary role in the β-oxidation of fatty acids, and regulates grain number and panicle primary branching [11]. Panicle primary branching is another important trait for increasing the grain yield by increasing the grain number. Identification of gene/QTLs for panicle primary branching and use in introgression along with other yield attributing gene/QTL is a good strategy to improve the total grain yield. *DEP1* (Dense and Erect Panicle) is a dominant regulator of panicle primary branching mapped on chromosome 9. It encodes a *Gγ* that is reported to control and regulates the panicle primary branching, grain number. Therefor targeting grain number and panicle branching will be an attractive strategy to increase the grain yield to mitigate the food scarcity. *DEP1* is one of the major QTL in *japonica* based super rice breeding pipeline in northern China. Similarly, QTL, *qGN4.1*, was identified on chromosome 4, co-located with the other QTLs for panicle branching, tiller number, flag leaf length and width [12]. This QTL was transferred into 12 mega varieties of rice as a result there was significant increase in the grain number per panicle. The base variety was having 21.6 grains per panicle whereas the improved version was having 147.2 grains per panicle. Currently, there are inclusion of more than 900 QTLs for grain number in the rice database at Gramene, but precise genic information underlying these QTLs remains largely unknown as only a few of them have been fine mapped/cloned [11].

The utility of the mapped QTLs has become straightforward in breeding, since the QTL linked markers, themselves can act as foreground markers in marker assisted selection (MAS). Parental line selection having wide phenotypic variation for the desired trait is the key to the success of a linkage-based mapping [13]. Despite the genetic divergence for grain number among *indica* and *japonica*, mapping the QTLs linked to this trait using *indica*/*japonica* cross combinations has remained scanty in the literature, which could be due to their poor cross compatibility [14, 15]. To make this gap up, in our study, we have chosen one *indica* parental line PR126 having a low grain number and a *japonica* derived line, Pusa NPT34 having a high grain number for mapping QTLs using SSR markers. Further, we have attempted to identify the putative candidate genes using in silico analysis.

## Materials and methods

### Plant material

In the current study PR126 which matures early, having less total grain number, shorter panicle, less primary branches per panicle, was used as a female parent whereas Pusa NPT34 having contrasting phenotype for the above said traits was used as male parent to develop recombinant inbred lines (Table 1). PR126 is a short duration *indica* rice variety matures in 120 days. Developed originally as Huanghuazhan (HHZ), PR126 was bred in China having the parentage of Fenghuazhan /Huangxinzhang. It was one among the most popular green super rice (GSR) varieties distributed across the world by the International Rice Research Institute. Being widely grown in the southern China, HHZ shows wide adaptation, high yielding potential, profuse tillering and tolerance to multiple stresses. The other parent, Pusa NPT 34 was an advanced breeding line, derived from new plant type (NPT) breeding, having high grain number, good panicle architecture, and panicle density.

Hybridization between PR126 and Pusa NPT34 was attempted during *kharif* 2017 and thirty-three F_1_ were grown at IARI-Regional Breeding and Genetics Research Centre (IARI-RBGRC), Aduthurai, Tamilnadu during *rabi* 2017-18. After checking the hybridity using RM8094 marker, single true F_1_ plant was selected to generate F_2_ population. The F_2_ was advanced till F_6_ without any selection bias during the Recombinant inbred line (RILs) development. The advancement of the RILs followed a shuttle breeding method where DEL was used during *kharif* season and ADT during *rabi* season of the same year. A total of 175 RILs along with parental lines and checks were used generate phenotypic data at different locations. The population was raised and tested under normal conditions by following recommended package of practice as per the ICAR handbook of agriculture.

### Phenotyping of recombinant inbred lines

Three sites, spread across India namely, DEL (New Delhi; 28°64’N; 77°15’ E; 220 m), KAR (Karnal; 29°70’ N; 76°99’ E; 200 m) and ADT (Aduthurai; 11°08’N; 79°47’ E; 200 m) were selected for the evaluation of RILs during *kharif* 2020. These sites are the part of the shuttle breeding chain of ICAR-IARI exclusively used for rice crop improvement, and represented diverse agroecology. The experiment was conducted under irrigated transplanted ecosystems at all the sites, and the nursery was raised on elevated beds for 21 days and then transplanted into well puddled field with a spacing of 20 × 15 cm. The experiments followed a common design, augmented randomised complete block (ARCB) design with five checks viz., PR126, Pusa NPT34, Pusa Basmati 1509, Rasi and PKF_8_-218. Each entry was transplanted in two rows having ten plants each and all the entries were planted continuously without line gap between the genotypes in each block. At physiological maturity, five plants were randomly tagged from each family and data was recorded on the targeted traits. The traits observed were plant height (PH), panicle length (PL), primary branch number (PBN), total grain number per panicle (TGN), Tiller number (TN), filled grain number (FGN) and five plant grain yield (YLD). The data on unfilled grains (UFG) and spikelet fertility (SF) were derived from above observations. The data on the grains were recorded after harvest of the tagged plants. The mean of five plants of each family is considered for further data analysis. Border plants were excluded to reduce the error.

### Linkage map construction

Genomic DNA was isolated from each RIL using freshly collected leaves from the field using CTAB method [4, 16]. After DNA isolation, 1083 SSR markers were used for parental polymorphism and the product were separated using 3.5% agarose gel electrophoresis. The amplified and resolved PCR amplicons on gel electrophoresis were classified as PR126 type (A), Pusa NPT34 type (B) and heterozygotes(H). The genotypic data was subjected to chi-square test, and the marker which shows segregation distortion, low-rate amplification was deleted. A linkage map was constructed using the software QTL ICIM v4.2 by multipoint analysis using the Kosambi mapping function and a LOD value of 3.0.

### Statistical testing

Analysis of variance (ANOVA) was performed for all the phenotypic data collected in the current study location wise as well as across the locations. Adjusted mean value (BLUPs) for each trait was calculated the REML approach using the *lme4* package integrated with the software PBTools v1.4 (IRRI, 2014). The analysis was based on a mixed linear model, where blocks are treated as a random effect and treatments are treated as fixed effects for obtaining the adjusted means or BLUPs. The phenotypic distribution of all the traits was visualised through box plots using SR Plots tool. The genetic advance (GA) and heritability (ANOVA based approach) was calculated using multi-location phenotypic data using *traitstat* package in R. Correlation coefficients were calculated and graphically visualised using the *corrplot* package. Principal component analysis of the nine yield related traits was performed using *prcomp* in R base. Biplots were drawn for major principal components using the *GGEBiplot* package. Principal components and its contributing traits were graphically visualised using Circos.

### QTL mapping

QTL mapping was carried out to identify the genomic regions underlying the nine traits studied in the current experiment. QTL mapping was performed using ICIMapping v3.2. Threshold LOD values was calculated by running permutation test of 1000 iterations at alpha value 0.05 for each trait separately. Forward regression was used with walk distance of 1 cM and probability of inclusion at 0.01. QTLs were classified as major QTLs having more than 10% PVE and as minor QTLs having less than 10% PVE. A genomic region or marker interval possess more than one QTL for different traits are considered as QTL hotspots [12, 13].

### In-silico analysis of consistent QTLs

In-silico analysis was performed for major QTL hotspots to identify the probable putative candidate genes. The marker sequence information was used to know the physical position of the markers on the chromosomes. The probably expressed genes present between the marker positions were downloaded from the Rice Annotation Project Database (RAP-DB). Annotated candidate genes were shortlisted that have already known functions with the target traits based on previous reports. The interrelationship between the putative candidate genes in the QTL hotspot region and their association with the traits was established using the *knetminer* (https://knetminer.com).

## Results

### Morphological evaluation and analysis of variance

**Fig. 1:**
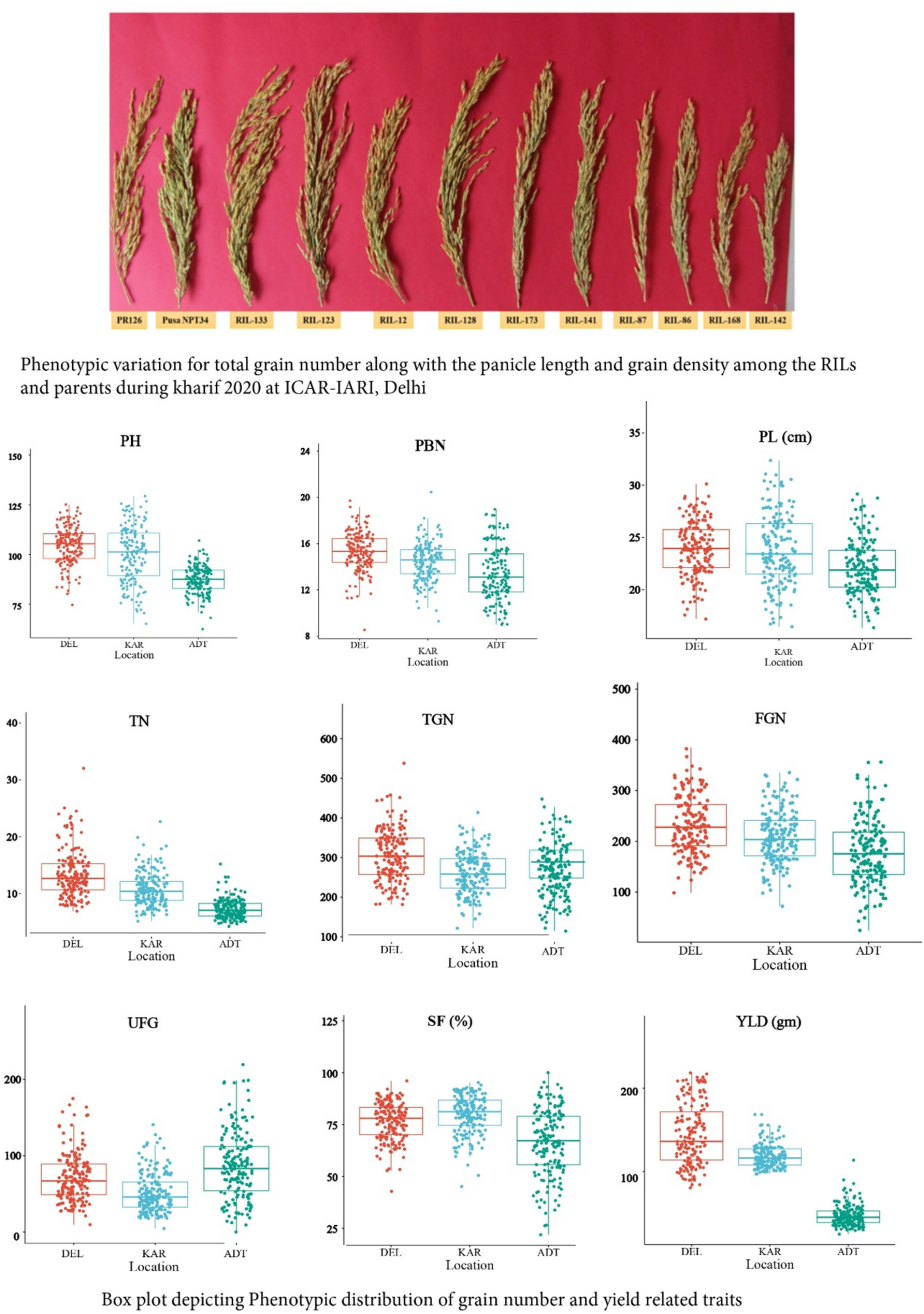
**A** Phenotypic variability for grain number and panicle related traits among the RILs during *kharif* 2020 at ICAR-IARI. **B** Box plot depicting the phenotypic distribution of grain number and yield attributing traits across three locations during *kharif* 2020. PH (Plant height), PBN (Primary branch number), PL (Panicle length), TN (Tiller number), TGN (Total grain number), FGN (Filled grain number), UFG (Unfilled grain number), Sf (Spikelet fertility), YLD (Yield)

The parental lines i.e. PR126 and Pusa NPT34 exhibited significant variation for the traits upon t-test. The mean performance of the parental lines is given Table 1. ANOVA revealed that all the traits exhibited highly significant variation across sites (Supplementary Table 1). Figure 1(a) illustrates that there is variation in TGN and other panicle-related traits across the RILs. The pooled ANOVA was performed to check for the presence of GE interactions and GxE interaction was also found highly significant (Supplementary Table 2).

**Table 1:** Descriptive statistics of PR126, Pusa NPT34 and RIL population across sites during *kharif* 2022

Traits	PR126	Pusa NPT34	Delhi			Karnal			Aduthurai		
			Range	Mean ± SE	CV	Range	Mean ± SE	CV	Range	Mean ± SE	CV
PH (cm)	90.02	100.58	74.73-125.09	104.58 ± 0.69	4.44	65.24-129.37	99.93 ± 1.04	6.44	62.53–107	87.40 ± 0.52	7.06
TN	18.59	12.59	6.65–31.79	13.18 ± 0.30	15.74	4.88–22.4	10.46 ± 0.26	13.09	3.95–14.95	7.06 ± 0.13	26.65
PL (cm)	22.07	18.96	17.05–29.97	23.79 ± 0.19	9.94	16.70-32.22	23.70 ± 0.26	7.90	16.21-29.00	21.97 ± 0.19	10.12
PBN	11.16	15.17	8.54–19.71	15.32 ± 0.12	5.29	9.29–20.45	14.43 ± 0.12	7.78	9.02–18.97	13.43 ± 0.17	7.61
FGN	176.75	301.53	98.51-385.13	233.64 ± 4.22	9.80	72.21-355.36	208.98 ± 3.90	24.09	24.54-355.31	176.98 ± 4.71	16.10
UFG	40.48	112.75	9.70–175.00	71.39 ± 2.39	25.58	4.63-140.68	51.56 ± 1.92	52.22	0-219.42	87.05 ± 3.30	24.02
TGN	209.63	433.06	181.45-538.31	305.02 ± 4.78	10.22	121.49-413.74	260.54 ± 3.99	20.91	114.21-447.78	264.03 ± 4.93	11.64
SF (%)	80.47	73.88	42.84–96.07	76.59 ± 0.67	4.10	45.17–95.25	79.93 ± 0.70	7.11	21.94–100	66.63 ± 1.19	6.25
YLD (gm)	142.22	160.23	80.43-218.47	162.11 ± 4.26	10.63	97.08-168.43	118.44 ± 0.87	14.74	24.83-113.63	47.25 ± 0.93	28.02

*NPT* New Plant Type, *CV* Coefficient of variation, *SE* Standard error, *FG* Filled grain, *PB* Primary branches, *PH* Plant height, *PL* Panicle length, *SF* Spikelet fertility, *TGN* Total grain, number, *TN* Tiller number, *UFG* Unfilled grain, *YLD* Yield

**Fig. 2:**
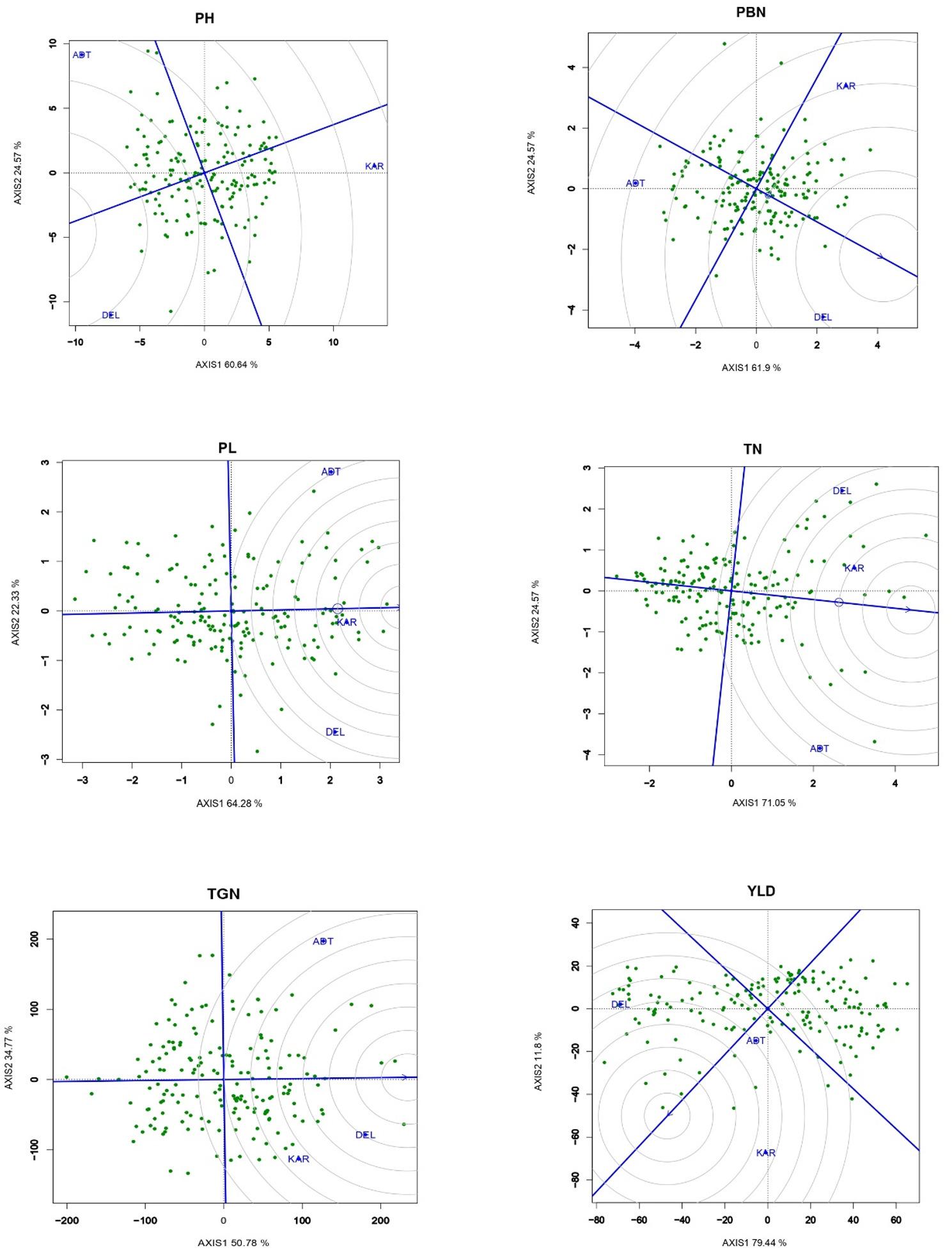
Ranking of the site performance for grain number and yield attributing traits during *kharif* 2020

The distribution pattern in box plot confirms the normal distribution pattern for all the traits and revealed that the traits PH, TN, PL, PBN, FGN, and TGN exhibited the highest mean performance at DEL, while the lowest performance was observed at ADT, except for TGN, which was low in KAR (Table 1. Figure 1(b), and Supplementary Table 3). The heritability (Broad sense, *H*^*2*^) was varied from as minimum as 11.66% and as high as 92.23%. The range confirms the wides range of heritability and presence of highly heritable as well as low heritable traits. The *H*^*2*^ of SF % at DEL was high whereas the lowest was observed for PL at KAR. Higher genetic advance was found for FGN, UFG, and TGN while, PL and TN had the lowest (Table 2). Ranking based on phenotype of the genotypes across the location indicated that mean performance of most of the traits of the RILs was higher at DEL, followed by KAR and ADT. DEL was found to be the best for PBN, PH, TGN with a high mean performance of the RILs. The population mean performance for TN, PL and YLD was observed to be good at KAR, followed by DEL and ADT. (Fig. 2).

**Table 2:** Genetic variability parameter for grain number and yield attributing traits at different locations

Genetic Parameter	BS h^2^ (%)			GA (%)
	Delhi	Karnal	Aduthurai	
PH (cm)	71.58	74.66	78.38	8.11
TN	75.00	74.33	70.70	0.91
PL (cm)	34.90	11.66	72.31	0.73
PB	81.23	76.03	56.81	1.22
FGN	78.23	83.91	58.00	20.87
UFG	79.89	76.10	55.00	20.21
TGN	78.06	78.62	65.00	30.00
SF (%)	92.23	88.67	63.55	5.50
YLD (gm)	35.02	43.79	52.91	5.90

### Interrelationship between the panicle architecture related traits

**Fig. 3:**
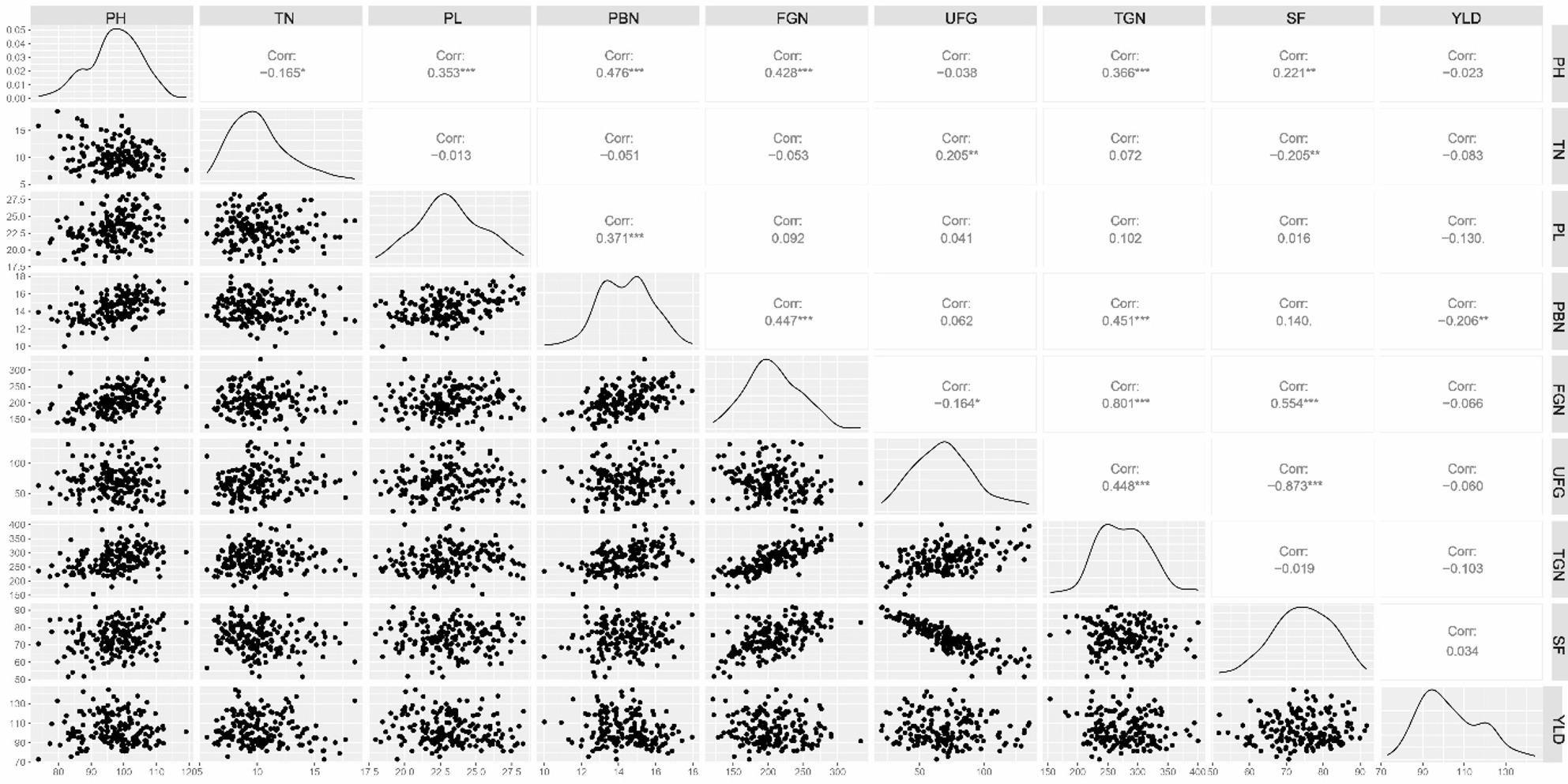
Correlation coefficient among the grain number and yield attributing traits using combined BLUP

**Fig. 4:**
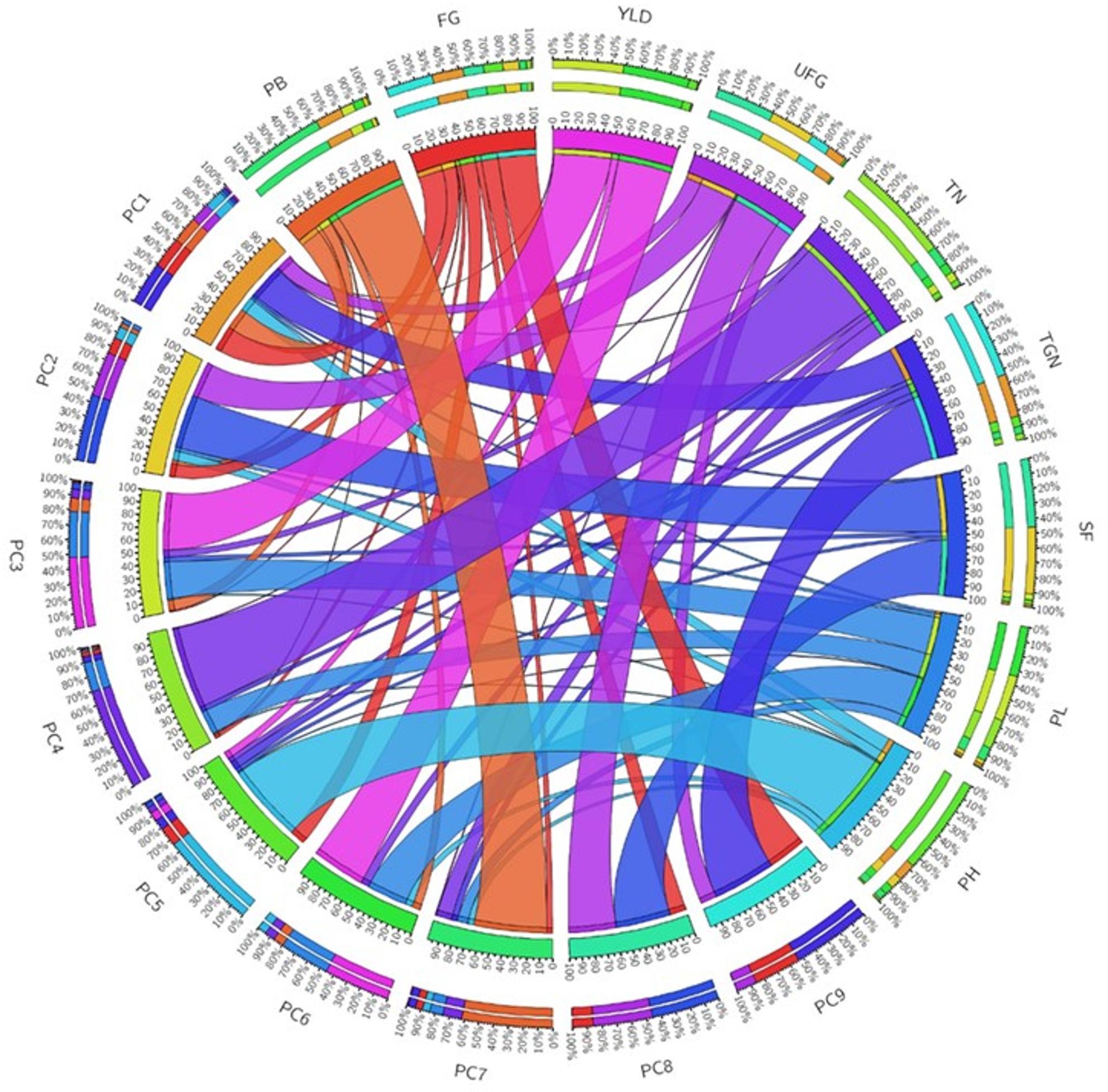
The contribution of different traits to principal components using combined BLUP

The correlogram showed that FGN, PBN, PH, PL, and TN were positively correlated with the TGN. Similarly, PH was positively correlated with PBN, FGN, SF, PL, but negatively correlated with the TN (Fig. 3). PBN was found positively correlated with TGN and PL. The principal component analysis showed four major principal components which explain a cumulative variance of 83% (Supplementary Table 4). The data visualisation using Circos Table Viewer v0.63-10 depicts the contribution of various traits on principal components (Fig. 4). The first principal component explains 33% of total variance followed by PC2 (21%), PC3 (16%), PC4 (13%). The major contributor to the total variance of PC1 was TGN, followed by FGN, PBN, UFG, PH similarly in PC2, major contributor was SF followed by FGN and PH, in PC3, major contributor was PBN followed by TN, PH, in PC4, major contributor to total variance was TGN, FGN, UFG. The TGN was highly variable followed by FGN, PBN and PH in the current study.

### Linkage map construction and QTL mapping

**Fig. 5:**
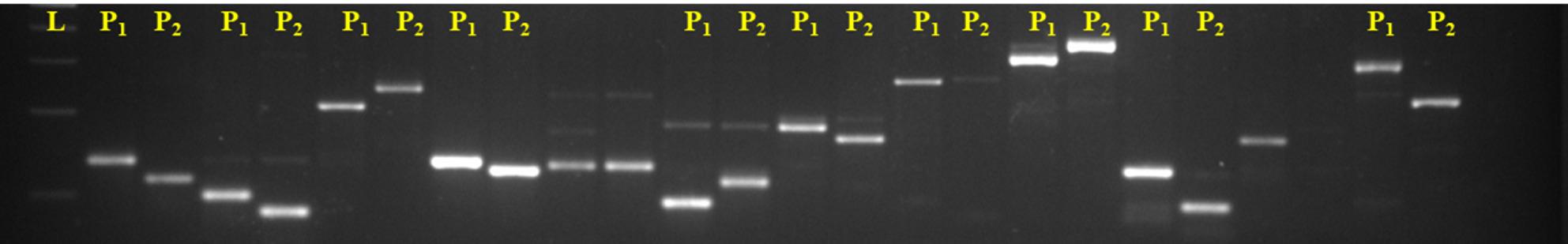
Representative gel picture of parental polymorphism between PR126 (P1) and Pusa NPT34 (P2)

**Fig. 6:**
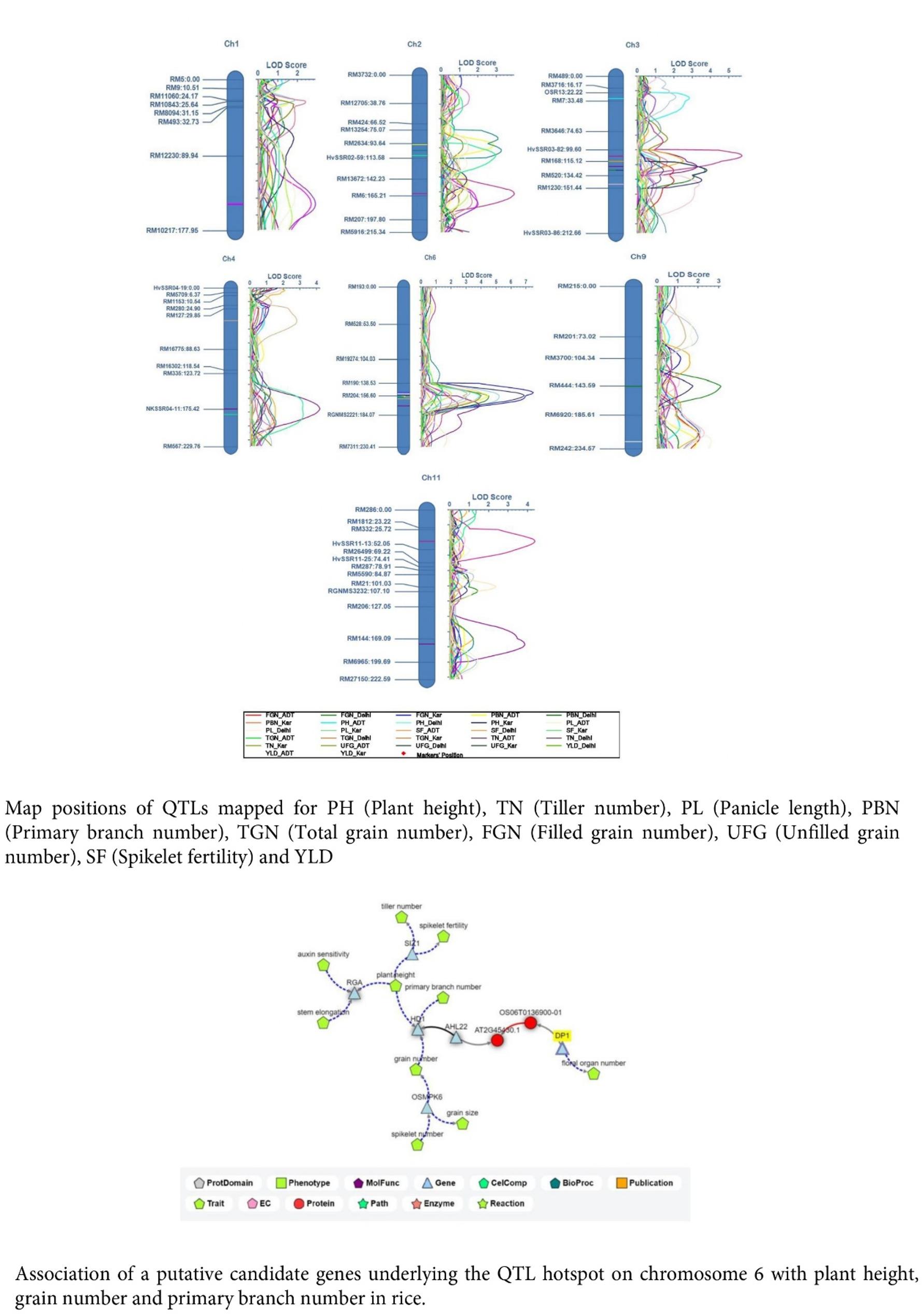
**A** Graphical representation of QTLs identified for various traits across the location. **B** Association of a putative candidate genes underlying the QTL hotspot on chromosome 6 with plant height, grain number and primary branch number in rice

Among the 1083 markers used for polymorphism survey, 11.63% of marker i.e. 126 markers were polymorphic. Out of 126 markers, 23 were removed as they were distorted. The list of polymorphic markers along with the physical position, forward and reverse primer details is given in (Fig. 5; Supplementary Table 5). These 103 markers record the genome diversity of 9.51% and the distribution of markers was uniform throughout the genome. The cumulative length of the complete genome was 2415.07 cM and average marker interval was 23.44 cM. The polymorphic markers distribution was ranged 5 on chromosome 7 & Chr 10 while 14 markers on chromosome 11 (Supplementary Table 6). The threshold LOD was calculated for each trait separately and given in Table 3. A total of 25 major and minor QTLs associated with all the traits and distributed on various chromosomes were identified (Table 3). Among them, seven QTLs were major QTLs and eighteen were minor QTLs (Fig. 6(a)).

**Table 3:** QTLs mapped for grain number and yield attributing traits in the RIL population of ‘PR126/PusaNPT34’

TQL name	Trait Name	Chromosome	Position (cM)	Left Marker	Right Marker	LOD**	PVE (%)	PVE (QTL x E)	Additive effect^#^
*qFGN3.1*	FG/Kar	3	32	OSR13	RM7	3.10 (2.87)	7.47	3.22	15.66
qFGN3.2*	FG/ADT	3	126	RM168	RM520	3.14 (2.89)	4.64	0.53	21.49
	FG/Delhi	3	127	RM168	RM520	3.69 (2.93)	10.38		20.02
qFGN6.1*	FG/Delhi	6	158	RM204	RGNMS2221	4.50 (2.93)	10.07	5.98	18.79
	FG/Kar	6	157	RM204	RGNMS2221	4.06 (2.87)	11.12		17.35
*qFGN9.1*	FG/Delhi	9	144	RM444	RM6920	3.09(2.93)	5.67	2.87	**14.82**
*qTGN3.1*	TGN/Kar	3	33	OSR13	RM7	3.45 (2.85)	4.61		16.47
qTGN3.2*	TGN/Delhi	3	127	RM168	RM520	4.82 (2.90)	11.67	1.13	28.71
qTGN6.1*	TGN/Delhi	6	156	RM190	RM204	6.94 (2.90)	10.58	3.90	27.44
	TGN/Kar	6	156	RM190	RM204	3.68 (2.85)	5.06		17.31
*qTGN11.1*	TGN/Kar	11	201	RM6965	RM27150	3.84(2.85)	5.56		18.03
*qPL2.1*	PL/Delhi	2	163	RM13672	RM6	3.58 (2.86)	5.80	0.28	0.75
qPL3.1*	PL/Delhi	3	108	HvSSR03-82	RM168	5.54 (2.86)	10.32	0.34	**1.00**
*qPL6.1*	PL/ADT	6	156	RM190	RM204	4.84 (2.89)	9.12		0.85
*qPL11.1*	PL/Delhi	11	43	RM332	HvSSR11-13	3.69 (2.86)	9.40	0.58	**0.97**
*qPH2.1*	PH/ADT	2	94	RM2634	HvSSR02-59	3.28 (2.97)	6.83	0.28	1.96
*qPH3.1*	PH/Delhi	3	144	RM520	RM1230	4.26 (2.92)	7.19	0.84	3.27
qPH6.1*	PH/Kar	6	159	RM204	RGNMS2221	4.86 (3.19)	12.77	1.49	5.21
	PH/ADT	6	157	RM204	RGNMS2221	4.66 (2.97)	9.90		2.36
*qPBN3.1*	PB/Kar	3	115	HvSSR03-82	RM168	4.03 (2.75)	6.73		0.54
*qPBN4.1*	PB/Delhi	4	175	RM567	nksssr04-11	4.20 (2.91)	5.18		**0.53**
qPBN6.1*	PB/ADT	6	152	RM190	RM204	7.47 (2.84)	18.27		1.17
*qPBN11.1*	PB/Delhi	11	176	RM144	RM6965	3.84(2.91)	7.34	0.26	**0.61**
*qTN2.1*	TN/Delhi	2	103	RM2634	HvSSR02-59	3.26 (2.61)	8.52	1.72	**1.51**
	TN/Kar	2	110	RM2634	HvSSR02-59	2.94 (2.85)	5.67		**0.89**
*qTN4.1*	TN/Kar	4	183	nksssr04-11	RM567	3.19 (2.85)	8.75	0.76	1.14
*qYLD4.1*	YPP/Kar	4	47	RM127	RM16775	2.92 (2.81)	6.38	0.62	**7.23**
*qYLD6.1*	YPP/Delhi	6	161	RM204	RGNMS2221	3.04 (2.95)	6.97		**23.92**
*qUFG6.1*	UFG/Delhi	6	155	RM190	RM204	3.52 (2.57)	5.20	1.18	12.93
*qSF1.1*	SF/Kar	1	147	RM12230	RM10217	2.89 (2.83)	7.34	0.03	**6.25**

*LOD* Logarithm of the odds, *RM* Rice microsatellite, *HvSSR* Highly variable simple sequence repeats

*major QTLs

**Threshold LOD

^#^Bold values=P1 and Unbold=P2

#### Filled grain number per panicle (FGN) and Total grain number (TGN)

Four QTLs were mapped for FGN namely, *qFGN3.1*,* qFGN3.2*,* qFGN6.1*, and *qFGN9.1*, on chromosomes 3, 6, and 9, respectively. The marker interval RM204 and RGNMS2221 possessed a QTL for FGN namely, *qFGN6.1.* A separate QTL mapping using data generated from DEL and KAR was able to detect the QTL in the same marker interval on chromosome 6. The LOD scores for this QTL ranged from 4.06 to 4.50. The PVE by *qFGN6.1* was 10.07% at DEL and 11.12% at KAR. Furthermore, the additive effect contributed by the Pusa NPT34 allele was 18.79 and 17.35 at the respective locations. Another QTL, *qFGN3.2*, which remained consistent at DEL and ADT was mapped between the markers RM168 and RM520 on chromosome 3. The PVE of the QTLs was ranged from 4.64% to 10.38%, with the additive effect of 20.02 and 21.49 due to Pusa NPT34 respectively. The remaining QTL for FGN namely *qFGN3.1*, and *qFGN9.1* were mapped on chromosomes 3 and 9 between OSR13-RM7 and RM444-RM6920, respectively. The genomic region between RM444 and RM6920 harbors *qFGN9.1*, a novel QTL for FGN. The LOD score of 3.09, explaining 5.67% of the PVE, with an additive effect of 14.82 attributed to the allele from PR126 (Table 2). Likewise, we have identified four QTLs for TGN on chromosomes 3, 6 and 11. Among four QTLs identified for TGN, *qTGN3.1* and *qTGN3.2* falls in the same marker interval as that of *qFGN3.1* and *qFGN3.2* (Table 3). The study was able to identify a consistent QTL, *qTGN6.1* across two locations.

#### Panicle Length (PL)

Four QTLs namely, *qPL2.1*,* qPL3.1*,* qPL6.1* and *qPL11.1* were mapped on chromosomes 2, 3, 6 and 11 respectively. The genomic region flanked by the markers HvSSR03-82 and RM168 contains a major QTL, *qPL3.1*. This QTL demonstrated a LOD score of 5.54, accounted for 10.32% of the Phenotypic Variance Explained (PVE), and showed an additive effect of 1 attributable to the allele present in the line PR126. The remaining QTLs were minor QTLs namely, *qPL2.1*,* qPL6.1*,* qPL11.1*, mapped between flanking markers, RM13672-RM6, RM190-RM204, RM332-HvSSR11-13, respectively (Table 3).

#### Plant Height (PH)

Three QTL, *qPH2.1*, *qPH3.1*, and *qPH6.1*, were mapped on chromosomes 2, 3, and 6, respectively. The major QTL, *qPH6.1*, was associated with the markers RM204 and RGNMS2221 and was detected at both KAR and ADT. This QTL showed LOD values ranging from 4.66 to 4.86, with a PVE of 9.90 at ADT and 12.77% at KAR, along with additive effects of 2.36 and 5.21 contributed by Pusa NPT34, respectively. In contrast, *qPH2.1* and *qPH3.1* were minor and site-specific QTLs. The QTL *qPH2.1* was mapped between RM2634–HvSSR02-59, while *qPH3.1* was mapped between RM520–RM1230 (Table 3**)**.

#### Primary Branches Number (PBN)

Four QTLs were mapped, *qPBN3.1*,* qPBN4.1*,* qPBN6.1*, and *qPBN11.1* on chromosomes 3, 4, 6, and 11 respectively. The novel QTL, *qPBN11.1* was mapped between, RM144 and RM6965 on chromosome 11. This QTL showed a LOD score of 3.84, explained 7.34% of the PVE, and exhibited an additive effect of 0.61 due to the allele inherited from the line PR126. The other marker interval RM190 and RM204 possessed a major QTL, *qPBN6.1* and *qPBN3.1*, a minor QTL, was mapped between HvSSR03-82-RM168 (Table 3).

#### Tiller Number (TN)

Two Quantitative Trait Loci (QTLs) associated with TN were identified on chromosomes 2 and 4, designated as *qTN2.1* and *qTN4.1*, respectively. The major QTL, *qTN2.1*, demonstrated high stability as it was consistently mapped at both the DEL and KAR sites, bracketed between the molecular markers RM2634 and HvSSR02-59. This QTL exhibited a favourable additive effect, ranging from 0.89 to 1.51 due to the PR126 parent, and accounted for 5.67% of the phenotypic variation explained (PVE) at KAR and 8.52% at DEL (with corresponding LOD scores of 2.94 and 3.26). In contrast, *qTN4.1* was identified as a minor, site-specific QTL found only at the KAR, mapped between the markers nksssr04-11 and RM567, and registered a LOD score of 3.19 (Table 3).

#### Yield (YLD)

Two minor, site-specific QTLs for YLD were mapped. QTL, *qYLD4.1* on chromosome 4 and *qYLD6.1* on chromosome 6. The first, *qYLD4.1*, was identified at the KAR site, flanked by RM127 and RM16775, contributing a PVE of 6.38% (LOD 2.92) with an additive effect of 7.23 from the PR126 parent. Conversely, *qYLD6.1* was specific to the DEL site, located between RM204 and RGNMS2221, showing a PVE of 6.97% (LOD 3.04) and a larger additive effect of 23.92, also originating from PR126. The study also reported additional QTLs for related traits, namely *qUFG6.1* and *qSF1.1* (Table 3).

### QTL hotspots

The current study was able to identify four QTL hotspots, where among 25 QTLs, 13 were distributed on different chromosome within these QTL hotspots (Table 4). Chromosome 2 reported to possess a QTL hotspot of size 1.65 Mb bracketed between RM2634 and HvSSR02-59 (Cluster I). The Second hotspot had four QTLs viz., *qTGN3.2*,* qPBN3.1*,* qPL3.1*,* qPH3.1* (Cluster II) mapped on the short arm of chromosome 3. This region was bracketed between the markers HvSSR03-82-RM1230 with marker interval length of 2.40 Mb. The long arm of chromosome 4 possesses a QTL hotspot bracketed between nksrssr04-11 and RM567 with total genomic size of 3.81 Mb. This QTL hotspot possesses two QTLs namely, *qPBN4.1* and *qTN4.1* (Cluster III). Fourth hotspot was identified on chromosome 6 which carries five QTLs namely, *qTGN6.1*,* qPL6.1*,* qPBN6.1*,* qPH6.1*,* qYLD6.1* (Cluster IV) and was found flanked between markers RM190 and RGNMS2221 with a span of 3.47 Mb.

**Table 4:** QTL hotspots identified in PR126 x Pusa NPT34 derived RILs during *kharif* 2020

Cluster number	Chromosome	Marker interval	Interval length (Mb)	Number of QTLs	Name of the QTLs
I	2	RM2634-HvSSR02-59	1.65	2	*qPH2.1*,* qTN2.1*
II	3	HvSSR03-82-RM1230	2.40	4	*qTGN3.2*,* qPBN3.1*,* qPL3.1*,* qPH3.1*
III	4	RM567-nksssr04-11	3.81	2	*qPBN4.1*,* qTN4.1*
IV	6	RM190-RGNMS2221	3.47	5	*qTGN6.1*,* qPL6.1*,* qPBN6.1*,* qPH6.1*,* qYLD6.1*

### In-silico analysis

QTL hotspot on chromosome 6 possess five QTLs for different traits and the total size of the marker interval is 3.47 Mb. The in-silico study of the QTL hotspot on chromosome 6 has showed that this region comprises 530 gene models. Among 530 genes, few genes which may have direct or indirect role in the biosynthetic pathway of the QTLs were identified. A total of 17 genes were putative candidates which may play a key role in regulation of grain number, plant panicle length and primary branching (Table 5). Four of which putative genes encode AP2 domain containing protein, two coded zinc finger proteins, two auxin responsive factors, and one gene each coding an AP1 complex subunit, *OsMAPK6*,* OsDP1*, F-box containing domains, MADS box family protein, helix loop helix protein, *WD-40*, G-protein coupled receptors and ethylene responsive elements.

**Table 5:** The predicted gene models in the *QTL* hotspot on chromosome 6

Locus ID	Start (bp)	Stop (bp)	Putative function
LOC/Os06g04540	1,962,306	1,963,580	*OsDepressed palea1*, expressed
LOC/Os06g06090	2,806,668	2,812,929	*OsMAPK6*, expressed
LOC/Os06g06750	3,162,801	3,169,415	*OsMADS5* - MADS-box family gene with MIKCc type-box, expressed
LOC/Os06g06870	3,250,462	3,261,364	zinc finger protein, putative, expressed
LOC/Os06g06900	3,273,373	3,276,890	helix-loop-helix DNA-binding domain containing protein, expressed
LOC/Os06g06970	3,310,866	3,311,822	*AP2* domain containing protein, expressed
LOC/Os06g07000	3,319,111	3,322,612	*OsFBL28*-F-box domain and LRR containing protein, expressed
LOC/Os06g07030	3,337,083	3,338,612	AP2 domain containing protein, expressed
LOC/Os06g07040	3,342,423	3,346,318	*OsIAA20* - Auxin-responsive Aux/IAA gene family member, expressed
LOC/Os06g07090	3,376,566	3,386,549	*AP-1* complex subunit gamma-1, putative, expressed
LOC/Os06g07540	3,632,663	3,639,659	WD-40 repeat family protein, putative, expressed
LOC/Os06g08340	4,040,908	4,041,551	*AP2* domain containing protein, expressed
LOC/Os06g08360	4,062,304	4,063,540	ethylene-responsive element-binding protein, putative, expressed
LOC/Os06g09310	4,678,838	4,680,332	zinc finger, C_3_HC_4_ type domain containing protein, expressed
LOC/Os06g09390	4,731,330	4,733,911	*AP2* domain containing protein, expressed
LOC/Os06g09660	4,926,492	4,932,177	auxin response factor, putative, expressed
LOC/Os06g09930	5,060,660	5,065,027	G protein coupled receptor, putative, expressed

## Discussion

During the 1990s, researchers at IRRI were propounding a novel model for rice plants known as the new plant type (NPT). Pusa NPT34, used in this study is a high yielding breeding line derived from the NPT linage and characteristically possessed high number of grains. In the next decade, the idea of breeding for GSR was initially floated in China in 2005, for assimilating the ‘green’ traits - traits that are environment friendly, impart resource use efficiency and multiple stress tolerance [17]. Several multiparent inter-cross lines were bred integrating semi-dwarf trait with super yield and grain quality. One among these was HHZ, a high yielding, widely adapted, early maturing and lodging resistant variety. Soon HHZ found itself a part of the GSR project and released as PR126 in Punjab, it proved highly adaptable and early maturing. Soon PR126 started replacing long-duration popular variety, Pusa 44 [17].

The biparental population of PR126/Pusa NPT34 that was stabilised and was in the F_6_ generation, we could demonstrate that the distribution of traits was normal and predominantly quantitative, enabling a perfect condition for QTL mapping. Broad-sense heritability for grain number and primary branching, while high, must be carefully validated, as it includes non-additive genetic variance also. These non-additive variances contribute to the genetic variance but do not reflect the true breeding value. Therefore, the high heritability suggests that a significant portion of the observed genetic variation may be due to combination of additive and non-additive variance. Higher heritability with more additive effect for the traits can be transferable through breeding into the elite varietal background to enhance the productivity [18]. Evaluation and pooled analysis of RILs across multiple location, reduces the variation and error produced due to uncontrollable factors like soil heterogeneity, minor weather fluctuations, pest pressure, or subtle field gradients. These highly localized, random environmental effects are essentially averaged out over the larger data set. By collecting data across many environments, the analysis focuses on the average genetic effect across all sites, while the random environmental noise and the main environment effect are separated and minimized, leading to a more precise estimate of the true genetic potential. The comparative analysis of RIL performance across the three test locations (DEL, KAR, and ADT) revealed substantial GEI, evidenced by the significant variation in mean performance for several key traits. Overall, the mean trait performance for the RIL population was highest at DEL, strongly suggesting that this location provided a superior, more conducive environment for the expression of favourable traits compared to KAR and ADT. Even though the GxE interaction is present, few consistent Quantitative Trait Loci (QTLs), were detected. It means the overall variation in the trait has two distinct genetic components. This means, the QTLs are having an additive effect that is largely stable across all environments. They contribute to the main genetic effect (G) and are responsible for the genotype being generally superior or inferior regardless of the environmental conditions.

The linkage map of PR126/ Pusa NPT34 was developed using SSR markers, that covered the whole genome. The parents showed a low level of marker diversity indicating a great deal of genomic similarity between the parental lines. The most apparent commonality between PR126 and Pusa NPT34 was their *indica* background, although a pedigree level comparison was not possible. Moreover, the percentage of marker outturn (9.5%) for map construction was well comparable with SNP systems, which stood at a range of 3.0 to 10% of the total markers. The conglomeration of genomic regions at different marker interval/hotspots indicated that these hotspots are the region possesses closely related genes involved in the biosynthetic pathway of grain number and its associated traits. It makes pyramiding of the haplotypes carrying the hotspot region relatively easier. The QTL hotspot possesses a greater number of QTLs (five QTLs) for different traits was identified on chromosome 6. QTLs associated were *qTGN6.1*, *qPL6.1*, *qPBN6.1*, *qPH6.1*, and *qYLD6.1*. There were also two functionally associated QTLs of TGN, namely, *qFGN6.1* and *qUFG6.1* found at this hotspot. It was further interesting to observe that the most desirable QTLs for TGN, PL, PBN and YLD came from Pusa NPT34, except for PH. This eventually showed that this hotspot associated with taller plants that are higher yielding with a greater number of grains. QTL hotspot with positive alleles for yield attributing traits will be an important genomic region for marker assisted introgression to improve the yield barrier. During 2017, identified QTL hotspot on chromosome 3 where *qnt3.1*, and *qnt3.2*, *qph3.1*, *qTGW3.4* for TN, PH, and thousand grain weight were mapped [19]. Similarly on chromosome 4, QTL hotspot possessing *qTGW4.1* and *qPPP4.2* for thousand grain weight and panicles per plant were mapped. Another study during 2020, identified QTLs for DFF (*qFD6.1*), PH (*qPHT6.1*), TN (*qTL6.1*), PL (*qPL6.1*), on chromosome 6 [20]. The currently identified genomic regions are away from the report by Donde et al., 2020 but this hotspot region was reported to possess genes/QTLs for FGN, TGN, PL, PH and PBN from the previous reports [21].

The hotspot on the short arm of chromosome 3 (Cluster II) having four QTLs was the next interesting candidate genomic region. Spanned 2.4 Mb long, harboured *qTGN3.2*, *qPBN3.1*, *qPL3.1* and *qPH3.1* along with one functionally associated QTL of TGN, namely, *qFGN3.2*. The most desirable traits contributing QTLs for TGN, PBN and PH came from Pusa NPT34 except for PL. Although the integration of both these hotspots can improve grain number, a chance of increased PH and reducing the PL cannot be avoided. In order to use the QTL hotspot on chromosome 3, the genomic region must be fine mapped to identify the closely associated marker with no linkage drag of increased plant height. The marker linked to *qTGN3.2* was also associated with two earlier reported QTLs, *gpp3.1* and *gpp3.2* describing grains per panicle [22]. There are earlier reports of QTLs associated with PL, PBN, and PH co-localised in this hotspot region [23]. A recent study on marker trait association for filled grain, have identified an MTA on chromosome 3 for filled grain number at 3.85 Mb [24]. Another study has also identified a QTL on chromosome 3 for yield per plant at 7.68 Mb along with association for grain length, width and thousand grain weight on the same chromosome [25].

The hotspot on chromosome 2 (Cluster I) harboured QTLs for PH and TN, and was not a direct contributor to grain number. The QTL for both PH and TN in this hotspot was also reported by previous reports [26]. A recent report has identified MTAs for GNP, PBN, PL and TN [25]. The MTA for TN falls significantly away from the QTL detected in the present study. Similarly, another study was able to map a QTL for plant height and internode length on chromosome 3 at 12.5 Mb whereas the QTL in the current study was mapped at 20 Mb region [27]. The remaining hotspot on chromosome 4 (Cluster III) carried QTLs for PBN and TN, of which *qPBN4.1* was derived from PR126 and *qTN4.1* from Pusa NPT34. The size of this region is 3.81 Mb and, therefore, we need to pick out recombinants of this hotspot to get the desirable effects of both the traits together. The QTLs identified in this hotspot for PBN and TN were reported earlier [12]. During 2023, it was confirmed that the results of previous report by Deshmukh et al. for the presence of QTL for tiller number on chromosome 4 [28]. Our study also confirms the presence of QTL in the same genomic region on chromosome 4. The QTLs on chromosome 3 were considered as a single QTL obtained from Pusa NPT34, as it was found associated only with the grain number traits, TGN and FGN. This location was previously reported to carry QTLs for grains per panicle, *gpp3.1* [22] or *qGn3.1* [12]. There were also report that identified the presence of MTAs for grain number on chromosome 3 at 0.5 Mb which is near telomere end [29].

Of the nine QTLs that lay outside the hotspot regions, *qFGN9.1* and *qPBN11.1* are novel as there are no previous reports of a QTL affecting these traits. The QTL, *qFGN9.1*, is mapped between a 5.92 to 7.00 Mb on the long arm of chromosome 11. Although there are past studies reporting QTLs for grain number on chromosome 9, such as *gpp9.1* falling near the 21.18 Mb region [30] and *GP9.1* falling near the 17.74 Mb region [31], these QTLs are reasonably far placed from the currently discovered ones. Similarly, in an earlier report, the genomic region which harbours *qPBN11.1* was reported associated with genes for panicle neck diameter [32], however, no reports were found on its role in primary branch number in rice. Previous studies have provided reports on the remaining five QTLs. The QTL locus *qTGN11.1* was identified in studies conducted by [33] and [34], while *qSF1.1* was documented by Mei [26], *qYLD4.1* by Lian et al., [35], *qPL2.1* by Zhang et al., [36], and *qPL11.1* by Cui et al., [37], each associated with specific traits.

Identification of putative candidate genes and landing closer to the gene of interest in many of the genetic study become easy with the advent of genome sequencing and gene annotation in rice [38]. The QTL hotspot identified on chromosome 6, possessed 17 putative candidate genes display significant functional attributes. This region was selected as it harboured QTLs for TGN, PH, PL, PBN, and YLD, particularly coming from Pusa NPT34. Though qFGN9.1 and *qPBN11.1* were novel QTL they were not selected for in-silico analysis, as the PVE (%) of both the QTL was less than 10% and it needs study further to explore more about its candidate gene underlying the trait. The 17 putative candidate gene models encode various proteins which play direct role in inflorescence and spikelet development. Notably, *APETALA* genes (AP1 and AP2) zinc finger proteins, MADS box family proteins, helix loop helix proteins, WD40, G-protein coupled receptors and ethylene receptors play pivotal roles in rice spikelet development. APETALA proteins and MADS box family proteins reported to play major role inflorescence development. A recessive mutant, reported on chromosome 3 is a homeotic mutant of MADS box gene, can affect inflorescence development [39]. Similarly, many of the MADS box genes come under *SEPALLATA*-like gene and are associated with floral organ identity, inflorescence and spikelet development [40]. Apart from AP and MADS box family genes, WD40 protein reported to play an important role in inflorescence development through the regulating the primary branching. The *ASP1* (*aberrant spikelet*) encodes a protein called, WD40. This protein is homologous to *TPL/TPR* (*topless/topless related*) of *Arabidopsis* and *REL2* (*romosa enhancer loci*) of maize regulates the panicle primary branching and spikelet development in rice [41]. One of the major QTL for branching *DEP1* (Dense and Erect Panicle) acts through G-protein to regulate the panicle primary branching, length of the panicle and grain number [42]. Most of the putative candidate genes present in the QTL hotspot region plays key role in regulating the panicle architecture through the control of panicle primary branching, length of the panicle, grain number and through the regulation of the plant hormone production. A functional interconnectivity of the putative genes present in the QTL hotspot was found with *HD1* being linked to the PBN, as well as *AHL22* and *DP1*, which affect FGN. The interrelationship between these genes and traits can be attributed to the colocalization of QTLs on chromosome 6 (Fig. 6 b).

## Conclusion

In the current study, the QTLs were mapped for grain number, panicle primary branching and yield attributed traits and identified two novel genomic regions for grain number and panicle primary branching. Out of 25 QTLs identified, 16 QTLs were distributed in 4 marker intervals (hotspots) on different chromosomes in the current study. These results provide precise genetic markers that can be utilized in Marker-Assisted Selection (MAS) programs to efficiently combine favourable alleles. Breeders can focus on transferring these hotspot regions and the novel QTLs into elite germplasm to develop varieties with increased grain number and improved panicle architecture, both critical components of high yield. The scope of future study may involve the fine mapping of the major QTLs to identify the more closely linked marker, development of gene based or functional markers and cloning to understand the molecular mechanism of the gene/QTLs. It also helps us to use these genes in breeding program for more precise marker assisted selection and transfer.

## Supplementary Information

Supplementary Material 1

## Data Availability

The data sets supporting the results of this article are included in the supplementary file.
