# Supplementary material for: Mapping novel QTLs associated with grain number and primary branching in rice using new plant type derived RILs
Source: BMC Plant Biol. 2026 Mar 10;26:709. doi: 10.1186/s12870-026-08481-2 (PMC13088633; doi:10.1186/s12870-026-08481-2)
Supplement: Supplementary file 1 — Supplementary Material 1 [file 12870_2026_8481_MOESM1_ESM.docx]

**Supplementary table 1: Analysis of variance for grain number and yield attributing traits in RILs during *kharif* 2020 across the sites**

| Delhi | | | | | | | | | | |
| --- | --- | --- | --- | --- | --- | --- | --- | --- | --- | --- |
| Source | *df* | FGN | PBN | PH | PL | SF | TGN | TN | UFG | YLD |
| Treatment | 179 | 6051.56 ** | 7.2 ** | 107.16 ** | 6.62 ** | 134.05 ** | 12185.01 ** | 18.93 ** | 2572.34 ** | 5394.62** |
| Check | 4 | 129237.74 ** | 164.59 ** | 1014.15 ** | 17.53 * | 1586.42 ** | 344307.56 ** | 46.47 ** | 52766.15 ** | 2359.53* |
| Test vs Check | 1 | 22964.16 ** | 181.56 ** | 553.08 ** | 5.87 ** | 1979.53 ** | 34905.36 ** | 130.39 ** | 1246.04 ** | 195677** |
| Test | 174 | 3122.5 ** | 2.58 ** | 83.75 ** | 6.37 ** | 90.05 ** | 4419.43 ** | 17.66 ** | 1426.09 ** | 4318.48** |
| Block | 7 | 1210.51 | 1.1 | 14.9 | 1.34 | 20.23 | 2313.77 | 1.36 | 787.67 | 253.51 |
| Residuals | 28 | 502.27 | 0.62 | 21.22 | 5.63 | 10.18 | 944.93 | 4.54 | 340.83 | 332.73 |
| Karnal | | | | | | | | | | |
| Source | *df* | FGN | PBN | PH | PL | SF | TGN | TN | UFG | YLD |
| Treatment | 179 | 6611.21 ** | 7.55 ** | 204.36 ** | 19.48 ** | 116.22 ** | 12896.95 ** | 29.81 ** | 2171.74 ** | 1453.92* |
| Check | 4 | 178697.83 ** | 152.23 ** | 843.96 ** | 137.63 ** | 1344.13 ** | 438733.12 ** | 396.75 ** | 58852.49 ** | 1349.83* |
| Test vs Check | 1 | 5677.66 ** | 272.97 ** | 188.97 * | 915.07 ** | 5.92 ** | 61584.37 ** | 2274.78 ** | 29861.39 ** | 222776** |
| Test | 174 | 2660.56 ** | 2.7 ** | 189.74 ** | 11.61 ** | 88.62 ** | 2827.8 ** | 8.47 ** | 709.6 ** | 182.54 |
| Block | 7 | 2345.45 | 1.69 | 37.89 | 1.52 | 30.22 | 4953.03 | 2.55 | 1891.7 | 250.52 |
| Residuals | 28 | 2594.81 | 1.17 | 41.02 | 3.22 | 32.3 | 3164.23 | 2.48 | 903.66 | 359.14 |
| Aduthurai | | | | | | | | | | |
| Source | *df* | FGN | PBN | PH | PL | SF | TGN | TN | UFG | YLD |
| Treatment | 179 | 6364.85 ** | 8.87 ** | 69.75 * | 19.92 ** | 335.53 ** | 10709.15 ** | 45.07 ** | 3034.75 ** | 965.99** |
| Check | 4 | 113007.39 ** | 140.04 ** | 1063.5 ** | 52.2 ** | 1420.43 ** | 293667.73 ** | 34.29 ** | 45759.05 ** | 1314.06* |
| Test vs Check | 1 | 11965.22 ** | 106.3 ** | 131.86 ** | 2213.77 ** | 11329.55 ** | 3287.46 ** | 7415.18 ** | 27797.67 ** | 134503** |
| Test | 174 | 3881.12 ** | 5.3 ** | 46.55 ** | 6.57 ** | 247.41 ** | 4247.01 ** | 2.96 ** | 1910.27 ** | 196.54 |
| Block | 7 | 231.96 | 0.97 | 28.33 | 0.13 | 26.84 | 541.91 | 2.06 | 658.37 | 97.35 |
| Residuals | 28 | 845.03 | 0.99 | 38.37 | 4.28 | 19.22 | 931.87 | 6.93 | 384.2 | 239.06 |

*, significant at 0.05; ** significant at 0.01; FGN, Filled grain number; PBN, Primary branches number; PH, Plant height; PL, Panicle length; SF, Spikelet fertility; TGN, Total grain number; TN, Tiller number; UFG, Unfilled grain; YLD, Yield

| Groups | FGN | UFG | TGN | SF | PH | PL | TN | PBN | YLD |
| --- | --- | --- | --- | --- | --- | --- | --- | --- | --- |
| RILs | 3398.03** | 1584.88** | 6619.08** | 43.63** | 107.03** | 5.78** | 4.98** | 3.57** | 273.34** |
| Site | 723.81** | 251.20** | 551.09** | 42.32** | 75.78** | 73.09** | 29.83** | 0.85** | 3373.34** |
| RILsxSite | 1169.88** | 444.12** | 1206.04** | 88.62** | 29.30** | 5.24** | 7.51** | 1.33** | 1108.89** |
| Residual | 1335.02 | 589.14 | 1695.89 | 21.62 | 34.29 | 3.05 | 3.29 | 1 | 373.22 |

**Supplementary table 2: Pooled ANOVA for grain number and yield attributing traits in RILs during *kharif* 2020 across the sites**

*, significant at 0.05; ** significant at 0.01; RILs, Recombinant inbred lines; FGN, Filled grain number; PBN, Primary branches number; PH, Plant height; PL, Panicle length; SF, Spikelet fertility; TGN, Total grain number; TN, Tiller number; UFG, Unfilled grain; YLD, Yield.

**Supplementary table 3: Mean performance of the RILs across the sites for PH, TN, PL, PBN and FGN**

| **RIL** | **PH (cm)** | | | **TN** | | | **PL (cm)** | | | **PBN** | | | **FGN** | | |
| --- | --- | --- | --- | --- | --- | --- | --- | --- | --- | --- | --- | --- | --- | --- | --- |
|  | **Delhi** | **Kar** | **ADT** | **Delhi** | **Kar** | **ADT** | **Delhi** | **Kar** | **ADT** | **Delhi** | **Kar** | **ADT** | **Delhi** | **Kar** | **ADT** |
| 1 | 88.80 | 89.67 | 84.40 | 10.00 | 8.73 | 6.40 | 19.18 | 18.78 | 22.22 | 13.89 | 14.80 | 13.78 | 166.30 | 154.87 | 185.27 |
| 2 | 105.20 | 98.39 | 86.40 | 9.00 | 6.34 | 5.20 | 20.34 | 21.88 | 22.00 | 13.67 | 13.20 | 12.00 | 198.20 | 140.88 | 150.56 |
| 3 | 96.20 | 77.31 | 82.80 | 7.40 | 7.34 | 8.20 | 17.05 | 18.42 | 20.54 | 14.00 | 16.80 | 13.89 | 179.34 | 164.25 | 191.18 |
| 4 | 94.20 | 81.27 | 78.80 | 9.00 | 8.57 | 5.40 | 21.29 | 19.66 | 17.89 | 14.44 | 15.80 | 9.02 | 156.60 | 191.63 | 95.51 |
| 5 | 104.40 | 82.65 | 87.20 | 12.20 | 9.46 | 5.60 | 18.89 | 18.76 | 19.89 | 13.44 | 12.80 | 10.56 | 227.00 | 222.13 | 256.12 |
| 6 | 85.90 | 78.47 | 88.60 | 11.20 | 10.89 | 6.60 | 20.89 | 23.65 | 21.22 | 13.00 | 15.00 | 10.00 | 123.60 | 103.88 | 182.01 |
| 7 | 116.30 | 115.24 | 92.20 | 11.00 | 6.93 | 5.20 | 24.06 | 28.09 | 22.11 | 18.22 | 16.00 | 14.67 | 273.40 | 213.75 | 274.74 |
| 8 | 101.40 | 106.01 | 92.60 | 11.00 | 8.88 | 6.00 | 25.44 | 23.20 | 23.56 | 16.11 | 16.11 | 15.22 | 175.97 | 203.00 | 205.52 |
| 9 | 107.72 | 94.63 | 84.80 | 11.00 | 8.23 | 5.40 | 24.77 | 26.43 | 21.44 | 14.78 | 12.83 | 11.56 | 200.80 | 207.38 | 218.82 |
| 10 | 96.84 | 104.51 | 80.80 | 8.80 | 8.11 | 5.20 | 20.44 | 24.92 | 23.63 | 16.78 | 13.80 | 12.78 | 194.30 | 191.67 | 180.03 |
| 11 | 101.48 | 98.49 | 90.80 | 17.40 | 14.34 | 8.60 | 23.33 | 19.88 | 20.67 | 13.56 | 13.20 | 11.78 | 229.41 | 179.89 | 191.92 |
| 12 | 97.24 | 93.48 | 78.20 | 12.00 | 8.51 | 5.40 | 25.43 | 21.33 | 22.28 | 14.11 | 13.60 | 11.44 | 214.60 | 167.00 | 200.65 |
| 13 | 114.70 | 103.87 | 85.80 | 12.00 | 9.49 | 6.00 | 26.01 | 22.30 | 20.44 | 14.78 | 14.80 | 11.33 | 266.40 | 199.56 | 120.85 |
| 14 | 74.73 | 72.29 | 86.80 | 12.80 | 10.35 | 6.80 | 21.79 | 20.72 | 22.33 | 14.00 | 13.80 | 11.56 | 169.50 | 218.11 | 201.37 |
| 15 | 103.06 | 101.37 | 77.00 | 14.60 | 10.66 | 6.60 | 23.23 | 24.09 | 20.44 | 16.40 | 15.60 | 12.00 | 171.80 | 193.56 | 138.42 |
| 16 | 87.60 | 65.24 | 79.20 | 7.60 | 6.03 | 5.20 | 20.84 | 20.49 | 22.00 | 15.22 | 15.20 | 13.00 | 180.10 | 164.89 | 203.01 |
| 17 | 85.52 | 87.41 | 80.20 | 16.20 | 11.32 | 7.80 | 21.54 | 22.02 | 23.74 | 12.83 | 12.83 | 13.42 | 231.84 | 204.00 | 110.10 |
| 18 | 114.80 | 103.71 | 86.00 | 15.00 | 11.58 | 5.40 | 27.93 | 32.22 | 24.74 | 17.04 | 15.40 | 15.54 | 225.90 | 196.78 | 116.89 |
| 19 | 122.10 | 117.91 | 96.60 | 14.00 | 8.68 | 6.00 | 26.34 | 21.20 | 22.63 | 16.22 | 14.80 | 18.56 | 258.90 | 244.13 | 137.03 |
| 20 | 110.70 | 119.63 | 85.60 | 9.60 | 7.57 | 6.20 | 21.67 | 18.35 | 21.80 | 14.22 | 13.40 | 11.78 | 256.30 | 156.78 | 307.03 |
| 21 | 97.32 | 104.64 | 75.60 | 17.40 | 10.99 | 5.60 | 21.64 | 22.39 | 18.56 | 14.78 | 14.20 | 10.22 | 212.60 | 180.25 | 170.98 |
| 22 | 112.16 | 97.68 | 86.00 | 10.00 | 9.90 | 7.80 | 26.82 | 29.48 | 21.65 | 18.33 | 17.20 | 15.42 | 342.70 | 175.38 | 128.06 |
| 23 | 97.58 | 112.75 | 85.00 | 14.80 | 8.30 | 4.60 | 25.41 | 25.44 | 27.85 | 16.06 | 14.00 | 15.42 | 269.40 | 173.22 | 155.76 |
| 24 | 100.80 | 87.64 | 92.20 | 10.00 | 8.80 | 7.80 | 22.01 | 24.94 | 19.83 | 16.22 | 15.60 | 11.25 | 230.20 | 151.78 | 245.28 |
| 25 | 110.60 | 124.01 | 96.80 | 11.60 | 11.43 | 7.20 | 25.74 | 26.27 | 22.79 | 15.39 | 15.40 | 13.78 | 164.50 | 209.67 | 144.43 |
| 26 | 100.20 | 87.72 | 91.80 | 8.00 | 8.07 | 5.60 | 27.07 | 27.87 | 25.67 | 16.44 | 16.00 | 13.11 | 206.60 | 266.22 | 170.12 |
| 27 | 110.20 | 78.93 | 62.53 | 8.00 | 8.31 | 6.00 | 27.33 | 22.78 | 17.24 | 14.44 | 12.60 | 10.11 | 189.10 | 121.67 | 143.64 |
| **RIL** | **PH (cm)** | | | **TN** | | | **PL (cm)** | | | **PBN** | | | **FGN** | | |
|  | **Delhi** | **Kar** | **ADT** | **Delhi** | **Kar** | **ADT** | **Delhi** | **Kar** | **ADT** | **Delhi** | **Kar** | **ADT** | **Delhi** | **Kar** | **ADT** |
| 28 | 94.80 | 87.04 | 77.40 | 8.40 | 7.92 | 7.80 | 20.17 | 18.86 | 21.02 | 14.00 | 13.60 | 11.78 | 189.40 | 162.56 | 282.67 |
| 29 | 96.40 | 85.47 | 76.40 | 7.60 | 8.71 | 7.00 | 18.67 | 20.56 | 20.22 | 16.33 | 14.40 | 10.56 | 206.50 | 175.00 | 223.65 |
| 30 | 104.40 | 102.10 | 86.60 | 10.60 | 6.79 | 5.80 | 20.23 | 19.85 | 23.75 | 15.67 | 16.80 | 17.42 | 258.40 | 233.67 | 260.66 |
| 31 | 96.80 | 72.42 | 78.08 | 10.40 | 7.14 | 6.36 | 25.80 | 25.85 | 22.75 | 13.06 | 13.06 | 15.51 | 155.30 | 132.43 | 140.32 |
| 32 | 118.40 | 125.52 | 92.00 | 9.80 | 8.74 | 5.60 | 24.79 | 22.83 | 26.17 | 14.89 | 14.89 | 12.31 | 286.60 | 270.44 | 268.86 |
| 33 | 118.80 | 97.81 | 92.00 | 14.40 | 9.58 | 5.00 | 26.19 | 30.40 | 23.68 | 14.67 | 13.60 | 14.22 | 253.20 | 260.22 | 163.36 |
| 34 | 111.20 | 99.20 | 82.60 | 9.40 | 6.29 | 5.80 | 20.48 | 19.27 | 19.44 | 14.89 | 15.40 | 9.11 | 226.00 | 202.33 | 214.10 |
| 35 | 117.50 | 95.82 | 97.80 | 12.40 | 6.90 | 3.95 | 25.12 | 29.79 | 27.74 | 19.17 | 18.20 | 16.51 | 290.40 | 170.78 | 250.81 |
| 36 | 103.60 | 113.74 | 95.60 | 9.60 | 8.37 | 5.80 | 27.83 | 26.52 | 25.32 | 16.78 | 14.80 | 14.53 | 311.70 | 275.00 | 230.87 |
| 37 | 98.20 | 95.60 | 92.20 | 9.40 | 8.93 | 6.00 | 22.12 | 21.13 | 18.98 | 16.89 | 14.80 | 12.56 | 270.10 | 232.83 | 132.71 |
| 38 | 111.40 | 119.91 | 101.80 | 7.80 | 7.28 | 4.60 | 23.69 | 19.79 | 23.44 | 16.17 | 13.60 | 11.67 | 247.30 | 162.11 | 143.38 |
| 39 | 101.92 | 106.09 | 88.60 | 22.20 | 13.97 | 6.20 | 27.73 | 30.16 | 24.37 | 15.89 | 15.89 | 14.13 | 195.50 | 190.11 | 75.29 |
| 40 | 105.10 | 119.67 | 87.60 | 15.20 | 11.32 | 7.40 | 23.36 | 22.40 | 19.74 | 16.22 | 13.80 | 17.42 | 236.30 | 243.88 | 87.81 |
| 41 | 112.00 | 111.36 | 80.80 | 23.00 | 15.15 | 5.60 | 25.18 | 21.84 | 24.63 | 14.33 | 14.60 | 12.33 | 226.60 | 203.33 | 205.09 |
| 42 | 100.80 | 90.31 | 88.00 | 21.40 | 15.76 | 8.40 | 22.52 | 23.91 | 24.75 | 15.56 | 16.00 | 16.47 | 253.70 | 147.00 | 128.88 |
| 43 | 110.70 | 87.56 | 86.00 | 12.60 | 7.71 | 5.20 | 26.48 | 28.56 | 29.00 | 15.78 | 15.00 | 18.46 | 249.10 | 223.44 | 171.00 |
| 44 | 98.30 | 83.90 | 102.40 | 9.00 | 6.58 | 5.40 | 23.09 | 22.63 | 20.86 | 16.56 | 15.60 | 11.89 | 252.40 | 199.33 | 211.05 |
| 45 | 101.90 | 119.19 | 90.40 | 13.00 | 11.56 | 6.80 | 26.93 | 26.77 | 24.76 | 16.78 | 15.00 | 17.34 | 298.50 | 298.25 | 195.89 |
| 46 | 97.50 | 107.19 | 81.47 | 11.60 | 9.94 | 7.24 | 24.67 | 20.04 | 22.10 | 14.22 | 13.40 | 16.43 | 227.80 | 143.17 | 220.75 |
| 47 | 105.20 | 111.79 | 88.40 | 8.80 | 5.84 | 4.80 | 23.88 | 24.85 | 22.07 | 14.78 | 15.60 | 13.00 | 127.90 | 195.56 | 210.84 |
| 48 | 105.74 | 100.89 | 98.00 | 8.40 | 7.56 | 5.20 | 22.74 | 21.98 | 23.06 | 15.56 | 15.20 | 15.11 | 193.50 | 184.33 | 198.84 |
| 49 | 102.80 | 84.02 | 92.00 | 9.40 | 6.78 | 5.40 | 23.79 | 24.74 | 19.56 | 13.56 | 14.40 | 9.78 | 196.20 | 164.44 | 235.51 |
| 50 | 95.16 | 88.30 | 91.00 | 6.65 | 7.63 | 8.00 | 19.89 | 25.84 | 21.68 | 13.44 | 14.00 | 17.63 | 186.10 | 183.67 | 229.15 |
| 51 | 106.24 | 92.47 | 86.20 | 8.60 | 6.11 | 4.60 | 22.61 | 21.90 | 20.00 | 14.56 | 14.40 | 11.44 | 245.70 | 245.89 | 42.71 |
| 52 | 105.90 | 122.41 | 91.60 | 10.20 | 9.38 | 8.20 | 22.77 | 24.59 | 20.11 | 14.67 | 14.00 | 13.89 | 242.70 | 288.25 | 150.55 |
| 53 | 83.54 | 94.70 | 71.00 | 11.40 | 9.32 | 7.60 | 22.03 | 24.20 | 20.52 | 11.89 | 11.40 | 10.67 | 98.51 | 286.33 | 123.57 |
| 54 | 88.56 | 101.31 | 91.20 | 11.20 | 11.74 | 8.60 | 22.18 | 24.89 | 22.97 | 11.67 | 12.00 | 12.44 | 207.00 | 274.75 | 120.73 |
| 55 | 98.26 | 103.58 | 92.00 | 11.60 | 10.21 | 8.00 | 28.18 | 26.15 | 23.50 | 15.22 | 13.60 | 13.11 | 195.30 | 241.67 | 72.31 |
| 56 | 91.58 | 79.06 | 88.40 | 7.80 | 6.09 | 6.00 | 22.90 | 22.59 | 21.28 | 13.67 | 12.80 | 13.11 | 193.90 | 212.56 | 197.57 |
| **RIL** | **PH (cm)** | | | **TN** | | | **PL (cm)** | | | **PBN** | | | **FGN** | | |
|  | **Delhi** | **Kar** | **ADT** | **Delhi** | **Kar** | **ADT** | **Delhi** | **Kar** | **ADT** | **Delhi** | **Kar** | **ADT** | **Delhi** | **Kar** | **ADT** |
| 57 | 116.40 | 112.37 | 90.80 | 12.00 | 12.24 | 7.60 | 24.18 | 19.50 | 20.03 | 14.56 | 13.40 | 11.89 | 291.70 | 233.89 | 175.80 |
| 58 | 108.54 | 121.87 | 100.40 | 9.20 | 5.99 | 4.80 | 25.81 | 27.76 | 26.26 | 14.44 | 16.40 | 15.52 | 217.60 | 240.89 | 257.07 |
| 59 | 101.60 | 78.86 | 82.80 | 12.60 | 11.63 | 6.40 | 23.07 | 17.97 | 19.59 | 14.00 | 11.00 | 13.22 | 147.40 | 194.11 | 24.54 |
| 60 | 106.66 | 84.45 | 93.80 | 7.80 | 7.22 | 4.80 | 25.46 | 25.07 | 23.06 | 14.89 | 12.00 | 15.11 | 219.10 | 234.22 | 158.90 |
| 61 | 108.72 | 122.36 | 89.00 | 8.80 | 9.72 | 7.20 | 24.68 | 24.03 | 27.53 | 17.33 | 13.80 | 16.52 | 366.80 | 199.00 | 253.66 |
| 63 | 94.84 | 104.51 | 91.80 | 11.80 | 9.71 | 5.80 | 21.74 | 24.73 | 22.79 | 12.89 | 12.89 | 14.00 | 168.00 | 190.75 | 148.89 |
| 64 | 114.74 | 96.54 | 87.60 | 13.60 | 10.61 | 7.00 | 25.26 | 30.00 | 28.43 | 16.56 | 13.80 | 14.41 | 233.60 | 188.71 | 136.23 |
| 65 | 107.50 | 93.95 | 92.20 | 7.80 | 6.55 | 6.40 | 23.89 | 24.98 | 24.55 | 15.11 | 14.80 | 17.43 | 300.40 | 254.33 | 182.76 |
| 66 | 108.68 | 97.27 | 82.60 | 11.20 | 8.36 | 5.60 | 26.44 | 24.39 | 19.89 | 15.33 | 15.40 | 13.22 | 239.27 | 226.00 | 282.36 |
| 67 | 101.50 | 104.34 | 79.20 | 16.60 | 12.40 | 7.80 | 24.63 | 25.74 | 20.26 | 15.11 | 12.47 | 11.78 | 241.99 | 220.88 | 182.45 |
| 68 | 103.25 | 105.90 | 89.00 | 31.79 | 14.39 | 7.00 | 23.53 | 27.70 | 21.78 | 14.33 | 12.20 | 12.78 | 144.50 | 253.33 | 210.55 |
| 69 | 113.60 | 105.77 | 85.25 | 17.40 | 15.38 | 12.64 | 25.61 | 28.46 | 21.33 | 15.89 | 15.80 | 17.43 | 325.30 | 240.57 | 175.38 |
| 70 | 109.46 | 113.12 | 80.20 | 11.80 | 10.56 | 6.80 | 23.63 | 24.39 | 20.50 | 16.56 | 16.20 | 13.78 | 312.20 | 308.78 | 128.18 |
| 71 | 99.90 | 91.90 | 80.50 | 13.60 | 8.11 | 4.50 | 23.72 | 25.12 | 20.00 | 12.67 | 12.60 | 11.89 | 168.97 | 155.99 | 215.62 |
| 72 | 107.10 | 108.20 | 73.75 | 12.40 | 9.13 | 6.00 | 28.79 | 26.33 | 16.89 | 15.78 | 14.76 | 9.00 | 245.60 | 275.88 | 103.30 |
| 73 | 101.70 | 112.05 | 80.00 | 13.00 | 9.48 | 8.60 | 23.79 | 27.13 | 20.66 | 15.33 | 14.60 | 14.56 | 287.34 | 255.38 | 220.93 |
| 74 | 101.20 | 76.20 | 84.00 | 16.00 | 11.55 | 5.00 | 26.49 | 29.78 | 24.45 | 16.56 | 14.20 | 17.74 | 250.60 | 207.00 | 201.71 |
| 75 | 109.50 | 106.36 | 91.00 | 13.20 | 11.39 | 5.60 | 21.54 | 26.12 | 23.87 | 14.56 | 15.00 | 11.89 | 280.40 | 253.22 | 201.57 |
| 76 | 97.64 | 79.14 | 85.80 | 13.20 | 9.50 | 9.60 | 21.94 | 19.90 | 20.22 | 15.00 | 14.80 | 10.89 | 158.84 | 167.11 | 180.71 |
| 77 | 100.10 | 108.54 | 80.20 | 12.00 | 9.60 | 4.80 | 26.30 | 23.36 | 19.98 | 15.89 | 14.40 | 13.11 | 168.80 | 174.89 | 114.84 |
| 78 | 108.30 | 101.80 | 91.20 | 11.40 | 10.68 | 7.40 | 23.98 | 19.50 | 22.39 | 17.22 | 14.00 | 12.67 | 218.90 | 242.33 | 130.38 |
| 79 | 120.60 | 103.48 | 90.20 | 11.80 | 6.93 | 4.80 | 26.79 | 24.64 | 18.88 | 15.22 | 12.20 | 12.22 | 312.02 | 224.75 | 184.39 |
| 80 | 107.20 | 96.76 | 93.60 | 16.00 | 10.18 | 6.40 | 27.97 | 26.79 | 22.60 | 15.00 | 13.00 | 13.11 | 275.44 | 159.67 | 140.60 |
| 81 | 115.20 | 121.70 | 95.25 | 13.60 | 11.53 | 8.36 | 27.28 | 25.97 | 25.33 | 16.67 | 16.60 | 18.97 | 229.80 | 137.00 | 235.38 |
| 82 | 113.80 | 103.35 | 93.60 | 10.40 | 9.72 | 5.80 | 26.17 | 23.05 | 20.89 | 13.44 | 13.00 | 10.22 | 188.70 | 168.83 | 158.55 |
| 83 | 99.20 | 84.38 | 88.40 | 16.20 | 13.82 | 7.60 | 24.78 | 27.65 | 23.88 | 11.44 | 12.80 | 10.44 | 151.87 | 97.89 | 112.32 |
| 84 | 109.80 | 102.67 | 93.00 | 12.00 | 10.44 | 6.20 | 20.61 | 17.66 | 19.89 | 13.67 | 14.20 | 12.22 | 222.50 | 168.67 | 157.30 |
| 85 | 118.60 | 114.87 | 79.37 | 7.80 | 8.83 | 8.46 | 27.00 | 28.37 | 24.22 | 15.40 | 15.00 | 12.22 | 225.20 | 192.78 | 277.45 |
| 86 | 92.40 | 98.44 | 85.40 | 12.80 | 11.08 | 5.40 | 22.94 | 21.96 | 18.96 | 15.50 | 13.40 | 12.78 | 204.50 | 146.00 | 160.76 |
| **RIL** | **PH (cm)** | | | **TN** | | | **PL (cm)** | | | **PBN** | | | **FGN** | | |
|  | **Delhi** | **Kar** | **ADT** | **Delhi** | **Kar** | **ADT** | **Delhi** | **Kar** | **ADT** | **Delhi** | **Kar** | **ADT** | **Delhi** | **Kar** | **ADT** |
| 87 | 107.80 | 92.09 | 89.20 | 13.80 | 9.88 | 5.80 | 23.83 | 20.92 | 21.46 | 17.00 | 12.60 | 12.00 | 174.40 | 168.13 | 124.55 |
| 88 | 113.40 | 106.40 | 93.20 | 11.20 | 10.20 | 6.40 | 28.56 | 26.05 | 24.53 | 17.40 | 16.80 | 16.52 | 348.11 | 192.56 | 330.52 |
| 89 | 97.00 | 111.88 | 83.20 | 12.40 | 12.08 | 7.60 | 22.56 | 23.28 | 20.07 | 14.10 | 13.00 | 12.22 | 187.40 | 114.17 | 130.54 |
| 90 | 106.50 | 97.82 | 92.80 | 11.60 | 8.84 | 5.00 | 24.64 | 26.33 | 20.88 | 15.40 | 12.80 | 11.78 | 274.74 | 255.50 | 230.48 |
| 91 | 111.00 | 118.90 | 91.58 | 15.20 | 12.61 | 10.47 | 24.61 | 20.09 | 21.74 | 15.33 | 13.80 | 16.34 | 230.47 | 137.78 | 305.82 |
| 92 | 106.00 | 96.74 | 94.20 | 9.00 | 7.74 | 5.40 | 27.00 | 27.09 | 22.64 | 14.56 | 15.20 | 16.43 | 177.70 | 149.33 | 238.20 |
| 93 | 103.80 | 85.68 | 93.00 | 8.80 | 9.08 | 8.20 | 18.67 | 16.56 | 19.71 | 15.78 | 15.40 | 14.33 | 228.92 | 214.00 | 141.79 |
| 94 | 96.80 | 86.08 | 89.40 | 7.60 | 4.88 | 4.40 | 22.33 | 21.04 | 23.98 | 13.00 | 14.88 | 12.63 | 138.92 | 122.76 | 123.49 |
| 95 | 119.40 | 99.82 | 99.20 | 8.20 | 8.76 | 7.00 | 28.08 | 30.26 | 26.63 | 17.80 | 15.60 | 16.32 | 200.80 | 119.11 | 232.92 |
| 96 | 105.40 | 109.97 | 93.80 | 8.20 | 5.94 | 5.80 | 25.58 | 30.16 | 23.97 | 16.78 | 15.80 | 12.22 | 191.10 | 164.14 | 252.15 |
| 97 | 97.80 | 96.07 | 90.20 | 9.40 | 7.97 | 6.40 | 26.89 | 28.28 | 21.72 | 17.44 | 15.00 | 13.38 | 291.30 | 189.88 | 277.31 |
| 98 | 121.10 | 129.37 | 107.00 | 8.80 | 8.06 | 6.20 | 28.58 | 21.90 | 22.44 | 19.71 | 17.20 | 14.78 | 268.70 | 211.56 | 267.84 |
| 99 | 104.40 | 101.30 | 83.20 | 14.40 | 13.29 | 8.40 | 21.06 | 20.18 | 18.22 | 14.22 | 14.60 | 10.11 | 175.40 | 180.78 | 93.87 |
| 100 | 106.70 | 80.25 | 89.00 | 15.40 | 13.75 | 7.00 | 22.06 | 22.47 | 19.29 | 15.22 | 14.40 | 12.11 | 227.16 | 186.00 | 62.42 |
| 101 | 96.30 | 111.55 | 82.68 | 13.20 | 10.14 | 8.26 | 24.94 | 24.56 | 21.33 | 14.33 | 11.89 | 10.78 | 191.50 | 185.86 | 355.31 |
| 102 | 125.09 | 110.75 | 85.20 | 15.00 | 9.71 | 6.20 | 21.08 | 22.67 | 16.21 | 16.89 | 15.89 | 13.40 | 382.30 | 261.11 | 356.18 |
| 103 | 95.10 | 78.36 | 86.00 | 13.00 | 8.13 | 6.80 | 25.02 | 22.88 | 20.50 | 15.56 | 11.67 | 11.22 | 190.67 | 167.57 | 204.90 |
| 104 | 109.90 | 101.04 | 97.80 | 8.40 | 9.40 | 7.60 | 24.83 | 27.77 | 22.75 | 15.67 | 15.89 | 16.41 | 167.90 | 287.75 | 182.41 |
| 105 | 117.60 | 101.30 | 77.40 | 15.40 | 10.32 | 8.00 | 22.01 | 22.68 | 24.10 | 17.67 | 13.44 | 18.51 | 283.80 | 269.13 | 274.82 |
| 106 | 99.20 | 84.88 | 97.60 | 12.54 | 10.01 | 8.00 | 23.72 | 30.83 | 25.82 | 15.44 | 12.56 | 17.53 | 204.40 | 214.75 | 49.40 |
| 107 | 118.90 | 90.18 | 92.20 | 15.64 | 10.12 | 7.80 | 21.73 | 27.64 | 28.62 | 15.00 | 11.78 | 13.43 | 263.80 | 218.25 | 170.79 |
| 108 | 115.80 | 117.19 | 86.20 | 10.52 | 10.19 | 5.80 | 26.52 | 24.59 | 22.63 | 17.89 | 12.11 | 15.52 | 300.77 | 191.38 | 151.39 |
| 109 | 116.60 | 85.02 | 83.80 | 13.63 | 12.64 | 6.60 | 22.53 | 18.59 | 20.53 | 14.67 | 14.67 | 10.00 | 198.78 | 301.33 | 321.95 |
| 110 | 106.30 | 87.48 | 90.20 | 11.63 | 10.66 | 7.80 | 23.63 | 24.29 | 20.93 | 12.33 | 10.44 | 10.00 | 180.42 | 123.89 | 191.72 |
| 111 | 123.60 | 100.51 | 93.60 | 11.80 | 8.33 | 6.80 | 24.27 | 21.70 | 18.78 | 18.39 | 20.45 | 11.89 | 319.94 | 307.33 | 217.17 |
| 112 | 105.32 | 104.31 | 85.40 | 9.20 | 8.99 | 5.40 | 23.72 | 18.39 | 19.27 | 16.22 | 17.44 | 11.38 | 322.40 | 289.11 | 218.63 |
| 113 | 94.36 | 92.11 | 76.40 | 12.80 | 11.30 | 7.60 | 19.02 | 16.70 | 18.14 | 17.10 | 15.00 | 12.00 | 249.01 | 202.33 | 196.09 |
| 114 | 90.60 | 80.82 | 74.00 | 13.60 | 12.52 | 8.60 | 21.36 | 16.32 | 17.67 | 11.30 | 9.29 | 9.44 | 159.20 | 165.33 | 120.85 |
| 115 | 92.80 | 92.61 | 85.00 | 11.40 | 8.60 | 6.60 | 23.57 | 26.52 | 21.17 | 12.30 | 13.20 | 12.33 | 185.10 | 72.21 | 140.93 |
| **RIL** | **PH (cm)** | | | **TN** | | | **PL (cm)** | | | **PBN** | | | **FGN** | | |
|  | **Delhi** | **Kar** | **ADT** | **Delhi** | **Kar** | **ADT** | **Delhi** | **Kar** | **ADT** | **Delhi** | **Kar** | **ADT** | **Delhi** | **Kar** | **ADT** |
| 116 | 113.36 | 89.58 | 91.80 | 13.00 | 11.03 | 6.60 | 21.58 | 17.07 | 18.00 | 14.94 | 15.20 | 10.89 | 249.30 | 206.00 | 309.80 |
| 117 | 97.14 | 110.67 | 80.80 | 12.20 | 10.52 | 8.60 | 23.97 | 22.99 | 21.78 | 13.61 | 14.60 | 11.89 | 251.99 | 221.56 | 108.57 |
| 118 | 107.30 | 111.04 | 85.80 | 15.60 | 10.64 | 7.40 | 23.39 | 26.40 | 19.89 | 11.30 | 17.20 | 11.78 | 304.56 | 202.44 | 181.51 |
| 119 | 109.12 | 102.71 | 79.60 | 20.00 | 15.22 | 7.80 | 27.64 | 21.56 | 18.67 | 17.10 | 15.20 | 11.78 | 201.45 | 223.89 | 113.39 |
| 120 | 97.80 | 74.39 | 79.20 | 18.40 | 15.39 | 7.40 | 24.33 | 22.90 | 22.33 | 15.39 | 16.40 | 12.56 | 303.00 | 299.20 | 270.53 |
| 121 | 100.50 | 96.53 | 86.00 | 12.40 | 9.17 | 9.00 | 22.06 | 17.21 | 17.78 | 14.44 | 15.40 | 10.22 | 211.70 | 247.56 | 196.30 |
| 122 | 109.90 | 109.42 | 88.20 | 10.80 | 8.57 | 6.80 | 24.33 | 20.70 | 18.89 | 13.67 | 16.80 | 11.33 | 214.89 | 198.00 | 170.62 |
| 123 | 107.00 | 109.73 | 86.40 | 11.00 | 8.88 | 8.40 | 25.67 | 22.14 | 21.46 | 16.44 | 15.60 | 13.22 | 385.13 | 223.13 | 219.47 |
| 125 | 110.70 | 123.93 | 91.60 | 11.60 | 7.81 | 6.40 | 21.50 | 22.63 | 20.26 | 16.22 | 15.60 | 13.56 | 324.10 | 335.36 | 148.63 |
| 126 | 119.50 | 111.41 | 86.80 | 20.50 | 12.11 | 6.60 | 25.11 | 20.69 | 21.60 | 17.78 | 14.40 | 15.33 | 219.30 | 274.67 | 150.70 |
| 127 | 106.70 | 108.77 | 85.40 | 17.80 | 12.03 | 9.00 | 20.44 | 20.60 | 19.03 | 14.44 | 13.40 | 12.22 | 239.30 | 171.60 | 154.62 |
| 128 | 121.75 | 114.49 | 93.00 | 15.50 | 12.58 | 7.20 | 29.97 | 26.21 | 23.25 | 16.78 | 13.00 | 15.00 | 315.90 | 288.33 | 70.69 |
| 129 | 98.50 | 82.49 | 81.20 | 21.75 | 18.33 | 7.80 | 23.61 | 23.06 | 19.72 | 15.00 | 12.20 | 13.44 | 254.00 | 210.00 | 207.59 |
| 131 | 110.67 | 100.80 | 86.80 | 23.33 | 18.12 | 6.00 | 25.71 | 23.48 | 26.94 | 14.94 | 15.79 | 16.53 | 289.10 | 256.99 | 180.51 |
| 132 | 104.88 | 90.81 | 88.40 | 24.25 | 14.04 | 6.60 | 24.39 | 19.91 | 20.56 | 15.67 | 14.76 | 12.22 | 237.30 | 216.87 | 189.43 |
| 133 | 99.18 | 101.82 | 83.48 | 11.40 | 11.43 | 7.36 | 27.31 | 21.94 | 25.93 | 17.00 | 15.40 | 15.32 | 233.00 | 314.63 | 200.87 |
| 134 | 87.80 | 70.55 | 83.00 | 21.20 | 14.25 | 6.40 | 24.54 | 23.77 | 21.78 | 15.78 | 13.60 | 12.22 | 318.40 | 233.44 | 200.04 |
| 136 | 83.60 | 76.01 | 79.40 | 24.80 | 22.40 | 8.40 | 23.82 | 25.44 | 23.83 | 14.44 | 11.80 | 12.44 | 181.60 | 163.44 | 71.78 |
| 137 | 97.48 | 99.59 | 92.40 | 20.00 | 14.54 | 6.00 | 19.89 | 19.14 | 22.06 | 16.56 | 14.60 | 13.44 | 315.30 | 259.67 | 100.33 |
| 139 | 106.25 | 83.49 | 75.80 | 23.75 | 15.91 | 7.60 | 21.99 | 22.19 | 17.33 | 14.61 | 12.67 | 10.56 | 209.62 | 194.98 | 155.78 |
| 140 | 108.90 | 91.68 | 78.20 | 14.80 | 12.82 | 6.80 | 21.40 | 22.84 | 19.46 | 15.67 | 14.60 | 12.33 | 216.30 | 181.33 | 193.04 |
| 141 | 101.30 | 113.23 | 80.20 | 14.60 | 11.49 | 8.40 | 24.19 | 28.40 | 21.91 | 16.50 | 15.60 | 12.44 | 233.40 | 230.00 | 130.53 |
| 142 | 110.40 | 89.18 | 85.40 | 10.00 | 8.46 | 7.80 | 19.78 | 22.81 | 21.28 | 15.30 | 14.00 | 14.67 | 137.70 | 206.43 | 107.15 |
| 143 | 106.50 | 100.76 | 89.20 | 11.40 | 10.48 | 6.40 | 20.53 | 19.30 | 19.78 | 15.60 | 17.60 | 11.56 | 176.87 | 162.56 | 238.44 |
| 144 | 108.20 | 121.10 | 86.20 | 10.00 | 11.64 | 8.80 | 23.67 | 23.53 | 22.33 | 17.22 | 16.60 | 10.56 | 311.75 | 289.63 | 67.97 |
| 145 | 96.90 | 114.69 | 89.20 | 13.20 | 9.22 | 5.80 | 21.92 | 23.31 | 21.47 | 16.50 | 12.40 | 12.67 | 155.00 | 143.14 | 221.00 |
| 146 | 96.10 | 116.92 | 98.60 | 10.40 | 7.50 | 5.60 | 23.39 | 21.71 | 22.67 | 16.22 | 17.60 | 12.78 | 181.90 | 247.67 | 173.34 |
| 147 | 109.70 | 99.92 | 91.40 | 11.00 | 8.81 | 7.40 | 28.78 | 25.75 | 19.73 | 15.67 | 12.40 | 14.22 | 288.94 | 165.44 | 250.17 |
| 148 | 102.63 | 112.29 | 87.72 | 12.64 | 16.56 | 14.95 | 27.63 | 30.92 | 23.83 | 18.44 | 15.80 | 16.42 | 239.70 | 330.38 | 140.86 |
| **RIL** | **PH (cm)** | | | **TN** | | | **PL (cm)** | | | **PBN** | | | **FGN** | | |
|  | **Delhi** | **Kar** | **ADT** | **Delhi** | **Kar** | **ADT** | **Delhi** | **Kar** | **ADT** | **Delhi** | **Kar** | **ADT** | **Delhi** | **Kar** | **ADT** |
| 149 | 114.64 | 115.97 | 93.80 | 17.74 | 11.32 | 9.00 | 21.61 | 20.06 | 25.62 | 16.67 | 13.40 | 18.44 | 286.28 | 313.50 | 187.84 |
| 150 | 110.97 | 106.16 | 95.00 | 15.56 | 9.82 | 6.20 | 24.73 | 23.21 | 21.73 | 15.00 | 13.40 | 17.33 | 220.40 | 231.33 | 260.47 |
| 151 | 107.64 | 86.41 | 86.20 | 11.60 | 8.57 | 8.60 | 23.97 | 26.70 | 20.11 | 14.33 | 14.20 | 12.67 | 209.40 | 128.43 | 155.09 |
| 152 | 113.40 | 101.44 | 95.60 | 13.20 | 10.27 | 9.60 | 25.72 | 20.45 | 20.87 | 15.33 | 14.80 | 15.00 | 299.10 | 148.00 | 132.84 |
| 153 | 107.50 | 126.71 | 95.80 | 13.20 | 11.49 | 5.60 | 24.17 | 21.35 | 25.76 | 16.00 | 14.78 | 14.43 | 221.90 | 201.87 | 152.78 |
| 154 | 103.70 | 91.64 | 90.60 | 13.00 | 10.11 | 9.20 | 25.11 | 22.61 | 24.61 | 13.11 | 12.60 | 12.89 | 268.50 | 112.00 | 180.22 |
| 155 | 95.80 | 105.35 | 97.60 | 14.60 | 11.45 | 10.00 | 27.36 | 26.92 | 23.31 | 15.67 | 15.20 | 14.33 | 289.02 | 222.00 | 119.61 |
| 156 | 88.60 | 94.00 | 74.80 | 21.62 | 19.62 | 10.40 | 23.64 | 22.49 | 19.61 | 18.33 | 15.20 | 16.45 | 315.80 | 124.67 | 160.48 |
| 157 | 104.62 | 118.84 | 91.52 | 12.87 | 10.92 | 8.71 | 24.62 | 23.58 | 21.71 | 17.22 | 15.80 | 15.63 | 285.09 | 229.70 | 137.63 |
| 158 | 109.70 | 110.05 | 94.40 | 15.43 | 12.84 | 7.60 | 22.63 | 30.27 | 26.71 | 16.00 | 15.20 | 14.65 | 308.98 | 187.56 | 157.47 |
| 159 | 117.60 | 122.96 | 95.80 | 12.74 | 10.58 | 8.20 | 21.53 | 27.07 | 21.80 | 14.33 | 16.20 | 13.89 | 329.90 | 151.57 | 324.59 |
| 160 | 106.74 | 103.76 | 96.52 | 13.76 | 12.85 | 11.73 | 24.63 | 22.15 | 22.63 | 16.00 | 14.00 | 14.56 | 306.80 | 281.22 | 120.64 |
| 161 | 81.80 | 71.44 | 68.20 | 21.83 | 15.76 | 10.00 | 18.73 | 22.08 | 17.62 | 16.44 | 14.48 | 10.78 | 209.70 | 221.46 | 87.39 |
| 162 | 103.70 | 104.35 | 80.80 | 11.53 | 7.61 | 6.00 | 25.73 | 27.78 | 21.94 | 14.17 | 15.55 | 15.33 | 148.09 | 177.13 | 231.98 |
| 163 | 114.38 | 111.62 | 84.00 | 14.75 | 12.99 | 9.00 | 20.74 | 24.43 | 21.11 | 17.94 | 16.78 | 11.44 | 339.70 | 321.72 | 98.46 |
| 164 | 107.30 | 107.18 | 83.60 | 10.63 | 6.97 | 5.00 | 23.61 | 23.29 | 20.52 | 17.44 | 16.20 | 15.43 | 320.67 | 215.11 | 277.60 |
| 165 | 96.90 | 86.25 | 78.40 | 17.63 | 14.36 | 8.60 | 21.61 | 22.68 | 25.53 | 15.67 | 13.80 | 14.43 | 199.56 | 197.00 | 141.65 |
| 166 | 113.30 | 98.85 | 86.80 | 14.53 | 14.33 | 8.60 | 23.62 | 28.16 | 24.13 | 13.22 | 13.60 | 13.00 | 252.38 | 231.52 | 166.23 |
| 167 | 104.70 | 112.75 | 83.00 | 11.62 | 10.52 | 6.20 | 21.61 | 22.92 | 26.72 | 15.00 | 16.52 | 16.73 | 221.46 | 201.42 | 141.52 |
| 168 | 118.10 | 102.48 | 87.80 | 15.26 | 12.15 | 8.60 | 24.82 | 27.23 | 22.92 | 8.54 | 15.00 | 12.43 | 156.68 | 212.56 | 110.09 |
| 169 | 87.30 | 94.55 | 79.80 | 22.64 | 17.62 | 10.20 | 22.87 | 21.89 | 20.99 | 14.44 | 10.20 | 9.89 | 227.31 | 193.33 | 95.93 |
| 170 | 96.40 | 79.98 | 89.80 | 17.53 | 10.59 | 7.60 | 21.84 | 22.53 | 20.06 | 13.78 | 12.00 | 13.22 | 149.40 | 328.56 | 185.72 |
| 171 | 108.00 | 87.04 | 85.00 | 14.65 | 12.35 | 8.00 | 23.72 | 25.91 | 22.52 | 16.44 | 14.51 | 15.44 | 263.24 | 233.77 | 245.32 |
| 172 | 115.70 | 111.79 | 94.00 | 12.54 | 10.19 | 8.00 | 25.83 | 29.82 | 27.51 | 15.56 | 13.52 | 17.44 | 210.20 | 221.76 | 157.07 |
| 173 | 109.90 | 124.44 | 90.40 | 17.83 | 13.75 | 7.80 | 17.43 | 21.14 | 20.90 | 16.78 | 15.40 | 16.44 | 227.90 | 224.38 | 163.31 |
| 174 | 103.30 | 98.52 | 81.60 | 19.62 | 12.62 | 7.00 | 22.63 | 20.76 | 20.34 | 17.22 | 15.20 | 13.67 | 205.10 | 221.56 | 140.21 |
| 175 | 105.86 | 108.18 | 85.80 | 10.40 | 11.29 | 9.00 | 17.99 | 21.57 | 19.73 | 15.17 | 14.60 | 17.53 | 270.60 | 299.11 | 205.76 |
| 176 | 106.34 | 106.36 | 95.51 | 16.00 | 12.53 | 12.65 | 26.54 | 27.72 | 23.63 | 15.00 | 17.42 | 13.43 | 186.40 | 201.76 | 103.52 |
| 177 | 104.34 | 117.04 | 100.00 | 13.60 | 10.09 | 5.60 | 24.89 | 25.30 | 26.88 | 13.33 | 14.40 | 14.53 | 271.35 | 289.33 | 73.40 |
| **RIL** | **PH (cm)** | | | **TN** | | | **PL (cm)** | | | **PBN** | | | **FGN** | | |
|  | **Delhi** | **Kar** | **ADT** | **Delhi** | **Kar** | **ADT** | **Delhi** | **Kar** | **ADT** | **Delhi** | **Kar** | **ADT** | **Delhi** | **Kar** | **ADT** |
| 178 | 108.60 | 105.71 | 94.20 | 10.20 | 9.19 | 7.80 | 24.36 | 25.44 | 21.36 | 14.67 | 12.80 | 14.44 | 255.10 | 243.11 | 177.29 |
| 179 | 99.60 | 83.19 | 78.20 | 15.20 | 13.56 | 8.20 | 22.91 | 24.18 | 18.89 | 16.56 | 13.20 | 9.56 | 301.10 | 233.89 | 142.28 |
| 180 | 80.20 | 75.60 | 93.00 | 14.40 | 15.74 | 11.80 | 26.28 | 20.50 | 21.22 | 15.33 | 12.20 | 12.33 | 162.38 | 181.78 | 90.51 |
|  |  |  |  |  |  |  |  |  |  |  |  |  |  |  |  |
|  |  |  |  |  |  |  |  |  |  |  |  |  |  |  |  |
|  |  |  |  |  |  |  |  |  |  |  |  |  |  |  |  |
|  |  |  |  |  |  |  |  |  |  |  |  |  |  |  |  |
|  |  |  |  |  |  |  |  |  |  |  |  |  |  |  |  |
|  |  |  |  |  |  |  |  |  |  |  |  |  |  |  |  |
|  |  |  |  |  |  |  |  |  |  |  |  |  |  |  |  |
|  |  |  |  |  |  |  |  |  |  |  |  |  |  |  |  |

**Supplementary table 3 (*Contd…*): Mean performance of the RILs across the sites for UFG, TGN, SF and YLD**

| **RIL** | **UFG** | | | **TGN** | | | **SF (%)** | | | **YLD (gm)** | | |
| --- | --- | --- | --- | --- | --- | --- | --- | --- | --- | --- | --- | --- |
|  | **Delhi** | **Kar** | **ADT** | **Delhi** | **Kar** | **ADT** | **Delhi** | **Kar** | **ADT** | **Delhi** | **Kar** | **ADT** |
| 1 | 55.33 | 41.90 | 69.18 | 221.63 | 196.77 | 254.45 | 75.03 | 78.71 | 72.81 | 198.47 | 148.67 | 79.94 |
| 2 | 68.88 | 89.50 | 43.84 | 267.08 | 230.38 | 194.40 | 74.21 | 61.15 | 77.45 | 134.00 | 105.53 | 41.60 |
| 3 | 76.00 | 75.75 | 57.62 | 255.34 | 240.00 | 248.80 | 70.24 | 68.44 | 76.84 | 123.55 | 125.73 | 53.93 |
| 4 | 75.13 | 90.63 | 60.57 | 231.73 | 282.26 | 156.08 | 67.58 | 67.89 | 61.19 | 131.73 | 110.63 | 47.46 |
| 5 | 28.00 | 22.25 | 12.30 | 255.00 | 244.38 | 268.42 | 89.02 | 90.90 | 95.42 | 156.24 | 131.78 | 34.50 |
| 6 | 76.67 | 114.00 | 146.04 | 200.27 | 217.88 | 328.05 | 61.72 | 45.17 | 55.48 | 149.20 | 109.64 | 42.46 |
| 7 | 39.22 | 117.13 | 35.54 | 312.62 | 330.88 | 310.28 | 87.45 | 64.60 | 88.55 | 140.54 | 106.23 | 43.84 |
| 8 | 65.00 | 55.88 | 68.84 | 240.97 | 258.88 | 274.36 | 73.03 | 78.41 | 74.91 | 102.14 | 118.33 | 32.81 |
| 9 | 108.44 | 83.00 | 75.94 | 309.24 | 290.38 | 294.76 | 64.93 | 71.42 | 74.24 | 170.32 | 128.79 | 38.40 |
| 10 | 47.44 | 31.11 | 96.92 | 241.74 | 222.78 | 276.95 | 80.38 | 86.04 | 65.00 | 187.12 | 134.29 | 48.83 |
| 11 | 45.22 | 50.44 | 80.55 | 274.63 | 230.33 | 272.47 | 83.53 | 78.10 | 70.44 | 189.76 | 144.41 | 77.76 |
| 12 | 71.22 | 56.00 | 45.54 | 285.82 | 223.00 | 246.19 | 75.08 | 74.89 | 81.50 | 160.25 | 168.43 | 39.16 |
| 13 | 67.78 | 79.44 | 76.97 | 334.18 | 279.00 | 197.82 | 79.72 | 71.53 | 61.09 | 213.45 | 116.12 | 59.54 |
| 14 | 40.78 | 45.44 | 90.77 | 210.28 | 263.55 | 292.14 | 80.61 | 82.76 | 68.93 | 159.42 | 114.75 | 53.30 |
| 15 | 36.33 | 42.44 | 77.85 | 208.13 | 236.00 | 216.27 | 82.54 | 82.02 | 64.00 | 217.08 | 113.63 | 46.80 |
| 16 | 30.78 | 42.22 | 27.85 | 210.88 | 207.11 | 230.86 | 85.40 | 79.61 | 87.94 | 123.55 | 100.92 | 42.93 |
| 17 | 39.83 | 35.33 | 70.06 | 271.67 | 239.33 | 180.16 | 85.34 | 85.24 | 61.11 | 150.18 | 117.26 | 46.31 |
| 18 | 35.11 | 26.56 | 26.77 | 261.01 | 223.34 | 143.66 | 86.55 | 88.11 | 81.37 | 102.53 | 133.9 | 30.44 |
| 19 | 49.44 | 40.00 | 68.64 | 308.34 | 284.13 | 205.67 | 83.97 | 85.92 | 66.63 | 108.40 | 115.37 | 44.50 |
| 20 | 34.11 | 26.56 | 0.00 | 290.41 | 183.34 | 315.44 | 88.25 | 85.51 | 100.00 | 164.87 | 105.45 | 36.84 |
| 21 | 53.67 | 44.50 | 31.26 | 266.27 | 224.75 | 202.24 | 79.84 | 80.20 | 84.54 | 200.74 | 122.22 | 39.34 |
| 22 | 108.89 | 67.00 | 185.63 | 451.59 | 242.38 | 313.69 | 75.89 | 72.36 | 40.82 | 161.56 | 115.05 | 45.41 |
| 23 | 62.33 | 81.67 | 69.90 | 331.73 | 254.89 | 225.66 | 81.21 | 67.96 | 69.02 | 198.94 | 125.17 | 30.92 |
| 24 | 79.00 | 63.56 | 107.14 | 309.20 | 215.34 | 352.42 | 74.45 | 70.48 | 69.60 | 181.24 | 107.67 | 45.11 |
| 25 | 89.67 | 47.56 | 70.80 | 254.17 | 257.23 | 215.23 | 64.72 | 81.51 | 67.10 | 152.62 | 112.16 | 47.87 |
| 26 | 48.22 | 33.78 | 112.12 | 254.82 | 300.00 | 282.24 | 81.08 | 88.74 | 60.27 | 140.29 | 111.26 | 43.41 |
| 27 | 50.67 | 76.11 | 89.42 | 239.77 | 197.78 | 233.06 | 78.87 | 61.52 | 61.63 | 96.33 | 97.08 | 34.25 |
| 28 | 67.00 | 30.00 | 49.60 | 256.40 | 192.56 | 332.27 | 73.87 | 84.42 | 85.07 | 198.47 | 111.67 | 56.29 |
| **RILs** | **UFG** | | | **TGN** | | | **SF (%)** | | | **YLD (gm)** | | |
|  | **Delhi** | **Kar** | **ADT** | **Delhi** | **Kar** | **ADT** | **Delhi** | **Kar** | **ADT** | **Delhi** | **Kar** | **ADT** |
| 29 | 51.33 | 39.88 | 75.09 | 257.83 | 214.88 | 298.74 | 80.09 | 81.44 | 74.86 | 131.75 | 103.22 | 50.53 |
| 30 | 33.75 | 36.67 | 35.27 | 292.15 | 270.34 | 295.93 | 88.45 | 86.44 | 88.08 | 135.13 | 104.79 | 44.80 |
| 31 | 42.33 | 47.14 | 35.62 | 197.63 | 179.57 | 175.94 | 78.58 | 73.75 | 79.75 | 178.57 | 111.15 | 63.79 |
| 32 | 78.78 | 34.67 | 20.66 | 365.38 | 305.11 | 289.52 | 78.44 | 88.64 | 92.86 | 166.59 | 123.42 | 30.85 |
| 33 | 80.00 | 49.22 | 52.58 | 333.20 | 309.44 | 215.94 | 75.99 | 84.09 | 75.65 | 119.43 | 102.98 | 35.50 |
| 34 | 37.38 | 51.44 | 53.39 | 263.38 | 253.77 | 267.49 | 85.81 | 79.73 | 80.04 | 150.36 | 110.31 | 29.29 |
| 35 | 58.75 | 27.44 | 18.62 | 349.15 | 198.22 | 269.43 | 83.17 | 86.16 | 93.09 | 128.17 | 129.51 | 29.77 |
| 36 | 46.11 | 23.67 | 45.93 | 357.81 | 298.67 | 276.80 | 87.11 | 92.07 | 83.41 | 141.28 | 117.57 | 70.51 |
| 37 | 41.78 | 77.50 | 112.15 | 311.88 | 310.33 | 244.86 | 86.60 | 75.03 | 54.20 | 218.47 | 140.64 | 73.82 |
| 38 | 27.44 | 42.44 | 25.01 | 274.74 | 204.55 | 168.39 | 90.01 | 79.25 | 85.15 | 127.71 | 105.95 | 38.35 |
| 39 | 72.22 | 47.56 | 92.68 | 267.72 | 237.67 | 167.97 | 73.02 | 79.99 | 44.82 | 144.02 | 132.8 | 33.27 |
| 40 | 52.78 | 25.63 | 112.48 | 289.08 | 269.51 | 200.29 | 81.74 | 90.49 | 43.84 | 128.24 | 103.56 | 45.19 |
| 41 | 120.89 | 48.22 | 38.78 | 347.49 | 251.55 | 243.87 | 65.21 | 80.83 | 84.10 | 108.78 | 129.46 | 32.08 |
| 42 | 58.00 | 41.33 | 125.38 | 311.70 | 188.33 | 254.26 | 81.39 | 78.05 | 50.69 | 135.40 | 105.09 | 44.28 |
| 43 | 35.88 | 20.00 | 82.68 | 284.98 | 243.44 | 253.68 | 87.41 | 91.78 | 67.41 | 104.91 | 125.21 | 90.03 |
| 44 | 50.44 | 44.00 | 160.03 | 302.84 | 243.33 | 371.08 | 83.34 | 81.92 | 56.87 | 194.40 | 105.06 | 45.82 |
| 45 | 77.00 | 25.25 | 90.74 | 375.50 | 323.50 | 286.63 | 79.49 | 92.19 | 68.34 | 106.67 | 118.28 | 39.87 |
| 46 | 75.33 | 45.17 | 123.46 | 303.13 | 188.34 | 344.21 | 75.15 | 76.02 | 64.13 | 158.16 | 137.66 | 64.36 |
| 47 | 54.86 | 37.56 | 85.60 | 182.76 | 233.12 | 296.44 | 69.98 | 83.89 | 71.12 | 160.88 | 128.57 | 49.44 |
| 48 | 100.33 | 25.56 | 48.94 | 293.83 | 209.89 | 247.78 | 65.85 | 87.82 | 80.25 | 136.82 | 123.85 | 55.82 |
| 49 | 57.11 | 45.98 | 68.29 | 253.31 | 210.42 | 303.80 | 77.45 | 78.15 | 77.52 | 140.01 | 105.12 | 41.27 |
| 50 | 109.78 | 56.11 | 60.84 | 295.88 | 239.78 | 289.99 | 62.90 | 76.60 | 79.02 | 124.11 | 123.42 | 52.42 |
| 51 | 50.67 | 49.67 | 78.92 | 296.37 | 295.56 | 121.63 | 82.90 | 83.19 | 35.11 | 158.16 | 122.22 | 24.83 |
| 52 | 73.33 | 25.00 | 109.63 | 316.03 | 313.25 | 260.18 | 76.80 | 92.02 | 57.86 | 135.46 | 109.88 | 50.80 |
| 53 | 65.89 | 22.00 | 135.08 | 182.09 | 308.33 | 258.65 | 63.81 | 92.86 | 47.77 | 117.96 | 127.49 | 34.40 |
| 54 | 79.44 | 22.25 | 89.00 | 286.44 | 297.00 | 209.73 | 72.27 | 92.51 | 57.56 | 130.73 | 108.43 | 39.05 |
| 55 | 60.00 | 48.89 | 98.30 | 255.30 | 290.56 | 170.61 | 76.50 | 83.17 | 42.38 | 117.12 | 115.53 | 41.05 |
| 56 | 27.56 | 18.00 | 52.72 | 221.46 | 230.56 | 250.29 | 87.56 | 92.19 | 78.94 | 148.17 | 112.92 | 42.60 |
| 57 | 59.44 | 25.44 | 48.43 | 351.14 | 259.33 | 224.23 | 83.07 | 90.19 | 78.40 | 134.70 | 104.4 | 38.00 |
| **RILs** | **UFG** | | | **TGN** | | | **SF (%)** | | | **YLD (gm)** | | |
|  | **Delhi** | **Kar** | **ADT** | **Delhi** | **Kar** | **ADT** | **Delhi** | **Kar** | **ADT** | **Delhi** | **Kar** | **ADT** |
| 58 | 57.33 | 40.89 | 48.48 | 274.93 | 281.78 | 305.55 | 79.15 | 85.49 | 84.13 | 152.25 | 109.89 | 45.27 |
| 59 | 85.89 | 81.78 | 104.19 | 233.29 | 275.89 | 136.52 | 63.18 | 70.36 | 21.94 | 195.39 | 115.02 | 45.05 |
| 60 | 100.56 | 52.22 | 92.48 | 319.66 | 286.44 | 251.38 | 68.54 | 81.77 | 63.21 | 161.79 | 107.92 | 33.76 |
| 61 | 48.78 | 54.50 | 60.03 | 415.58 | 253.50 | 313.69 | 88.26 | 78.50 | 80.86 | 100.09 | 127.27 | 49.95 |
| 63 | 50.50 | 49.75 | 28.34 | 218.50 | 240.50 | 177.23 | 76.89 | 79.31 | 84.01 | 147.24 | 109.87 | 38.31 |
| 64 | 53.33 | 35.57 | 14.22 | 286.93 | 224.28 | 150.45 | 81.41 | 84.14 | 90.55 | 121.56 | 111.3 | 34.68 |
| 65 | 68.44 | 53.11 | 65.60 | 368.84 | 307.44 | 248.36 | 81.44 | 82.73 | 73.59 | 152.41 | 108.97 | 39.97 |
| 66 | 99.33 | 40.80 | 35.55 | 338.60 | 266.80 | 317.91 | 70.66 | 84.71 | 88.82 | 127.40 | 106.5 | 41.08 |
| 67 | 56.11 | 71.89 | 108.95 | 298.10 | 292.77 | 291.40 | 81.18 | 75.44 | 62.61 | 207.19 | 103.8 | 35.53 |
| 68 | 34.67 | 24.00 | 70.91 | 181.45 | 277.33 | 281.46 | 80.65 | 91.35 | 74.81 | 135.18 | 102.17 | 42.29 |
| 69 | 47.22 | 26.29 | 82.84 | 372.52 | 266.86 | 258.22 | 87.32 | 90.15 | 67.92 | 132.81 | 115.02 | 74.56 |
| 70 | 85.44 | 50.89 | 156.70 | 397.64 | 359.67 | 284.88 | 78.51 | 85.85 | 44.99 | 110.72 | 99.21 | 40.77 |
| 71 | 50.89 | 43.54 | 83.27 | 219.86 | 199.53 | 298.89 | 76.85 | 78.18 | 72.14 | 174.68 | 117.71 | 64.39 |
| 72 | 89.67 | 78.38 | 219.42 | 335.27 | 354.26 | 305.65 | 73.25 | 77.88 | 33.80 | 104.71 | 119.02 | 36.15 |
| 73 | 52.22 | 45.88 | 122.17 | 339.56 | 301.26 | 343.10 | 84.62 | 84.77 | 64.39 | 173.00 | 141.53 | 85.56 |
| 74 | 98.40 | 44.63 | 76.43 | 349.00 | 251.63 | 278.14 | 71.81 | 82.26 | 72.52 | 94.11 | 119.16 | 31.30 |
| 75 | 67.22 | 47.33 | 45.71 | 347.62 | 300.55 | 247.28 | 80.66 | 84.25 | 81.51 | 188.30 | 101.13 | 51.13 |
| 76 | 64.88 | 56.22 | 95.10 | 223.72 | 223.33 | 275.81 | 71.00 | 74.83 | 65.52 | 152.88 | 117.24 | 54.50 |
| 77 | 91.75 | 57.56 | 96.17 | 260.55 | 232.45 | 211.01 | 64.79 | 75.24 | 54.42 | 128.79 | 127.26 | 30.98 |
| 78 | 129.53 | 127.56 | 120.71 | 348.43 | 369.89 | 251.09 | 62.83 | 65.51 | 51.93 | 94.63 | 135.31 | 49.57 |
| 79 | 81.67 | 32.63 | 150.74 | 393.69 | 257.38 | 335.13 | 79.26 | 87.32 | 55.02 | 207.37 | 128.45 | 33.42 |
| 80 | 56.22 | 38.11 | 68.74 | 331.66 | 197.78 | 209.34 | 83.05 | 80.73 | 67.16 | 122.82 | 102.26 | 52.66 |
| 81 | 95.11 | 53.50 | 135.71 | 324.91 | 190.50 | 371.09 | 70.73 | 71.92 | 63.43 | 91.64 | 128.39 | 56.27 |
| 82 | 28.22 | 21.67 | 30.38 | 216.92 | 190.50 | 188.93 | 86.99 | 88.62 | 83.92 | 210.09 | 100.16 | 49.37 |
| 83 | 43.89 | 38.44 | 32.64 | 195.76 | 121.49 | 144.96 | 77.58 | 71.80 | 77.48 | 193.29 | 168.43 | 42.32 |
| 84 | 100.22 | 73.11 | 85.95 | 322.72 | 241.78 | 243.25 | 68.95 | 69.76 | 64.67 | 162.87 | 126.34 | 57.47 |
| 85 | 45.00 | 29.22 | 86.43 | 270.20 | 222.00 | 363.88 | 83.35 | 86.84 | 76.25 | 181.94 | 153.65 | 77.83 |
| 86 | 64.56 | 33.75 | 95.68 | 269.06 | 179.75 | 256.44 | 76.01 | 81.22 | 62.69 | 201.46 | 139.13 | 34.55 |
| 87 | 21.22 | 20.88 | 30.10 | 195.62 | 189.01 | 154.65 | 89.15 | 88.95 | 80.54 | 212.82 | 113.67 | 37.48 |
| **RILs** | **UFG** | | | **TGN** | | | **SF (%)** | | | **YLD (gm)** | | |
|  | **Delhi** | **Kar** | **ADT** | **Delhi** | **Kar** | **ADT** | **Delhi** | **Kar** | **ADT** | **Delhi** | **Kar** | **ADT** |
| 88 | 69.78 | 47.56 | 97.31 | 417.89 | 240.12 | 427.83 | 83.30 | 80.19 | 77.25 | 147.14 | 105.16 | 44.14 |
| 89 | 98.78 | 40.17 | 120.34 | 286.18 | 154.34 | 250.88 | 65.48 | 73.97 | 52.03 | 127.00 | 116.98 | 58.80 |
| 90 | 61.56 | 42.25 | 18.60 | 336.30 | 297.75 | 249.08 | 81.69 | 85.81 | 92.53 | 168.90 | 107.1 | 54.60 |
| 91 | 9.70 | 19.89 | 91.61 | 246.80 | 157.67 | 397.43 | 96.07 | 87.39 | 76.95 | 179.34 | 100.91 | 113.63 |
| 92 | 33.78 | 42.78 | 117.66 | 211.48 | 192.11 | 355.86 | 84.03 | 77.73 | 66.94 | 126.75 | 124.04 | 35.51 |
| 93 | 27.78 | 32.67 | 85.87 | 256.70 | 246.67 | 227.66 | 89.18 | 86.76 | 62.28 | 97.97 | 129.61 | 45.15 |
| 94 | 80.40 | 56.87 | 198.15 | 219.32 | 179.63 | 321.64 | 63.34 | 68.34 | 38.39 | 120.87 | 131.5 | 34.47 |
| 95 | 39.63 | 80.56 | 92.20 | 240.43 | 199.67 | 325.12 | 83.52 | 59.65 | 71.64 | 195.34 | 115.28 | 55.57 |
| 96 | 82.89 | 90.71 | 50.84 | 273.99 | 254.85 | 302.99 | 69.75 | 64.41 | 83.22 | 110.41 | 107.73 | 46.04 |
| 97 | 44.22 | 42.75 | 129.73 | 335.52 | 232.63 | 407.04 | 86.82 | 81.62 | 68.13 | 126.70 | 124.08 | 38.68 |
| 98 | 54.88 | 32.67 | 71.70 | 323.58 | 244.23 | 339.54 | 83.04 | 86.62 | 78.88 | 120.95 | 142.49 | 40.48 |
| 99 | 49.44 | 38.22 | 128.19 | 224.84 | 219.00 | 222.06 | 78.01 | 82.55 | 42.27 | 189.87 | 119.15 | 47.61 |
| 100 | 63.56 | 52.99 | 98.95 | 290.72 | 238.99 | 161.37 | 78.14 | 77.83 | 38.68 | 167.99 | 111.29 | 49.90 |
| 101 | 47.63 | 41.88 | 25.24 | 239.13 | 227.74 | 203.86 | 80.08 | 81.61 | 87.62 | 149.23 | 155.41 | 51.77 |
| 102 | 72.38 | 81.88 | 46.82 | 454.68 | 342.99 | 403.00 | 84.08 | 76.13 | 88.38 | 111.61 | 129.4 | 34.27 |
| 103 | 58.31 | 41.29 | 24.08 | 248.98 | 208.86 | 228.98 | 76.58 | 80.23 | 89.48 | 103.49 | 111.59 | 37.00 |
| 104 | 95.89 | 31.38 | 88.15 | 263.79 | 319.13 | 270.56 | 63.65 | 90.17 | 67.42 | 129.76 | 99.72 | 47.83 |
| 105 | 175.00 | 47.13 | 110.94 | 457.86 | 316.26 | 385.76 | 61.98 | 85.10 | 71.24 | 110.67 | 145.74 | 36.13 |
| 106 | 77.78 | 57.13 | 138.65 | 282.18 | 271.88 | 188.05 | 72.44 | 78.99 | 26.27 | 133.43 | 101.17 | 50.56 |
| 107 | 105.44 | 20.75 | 88.75 | 369.24 | 239.00 | 259.54 | 71.44 | 91.32 | 65.80 | 116.16 | 156.15 | 40.10 |
| 108 | 42.67 | 18.00 | 75.98 | 343.44 | 209.38 | 227.37 | 87.58 | 91.40 | 66.58 | 108.57 | 115.19 | 37.15 |
| 109 | 67.11 | 27.33 | 37.86 | 265.89 | 328.66 | 359.81 | 74.76 | 91.68 | 89.48 | 207.95 | 112.22 | 64.35 |
| 110 | 49.22 | 34.56 | 198.83 | 229.64 | 158.45 | 390.55 | 78.57 | 78.19 | 49.09 | 176.51 | 113.02 | 42.52 |
| 111 | 59.78 | 29.75 | 111.02 | 379.72 | 337.08 | 328.19 | 84.26 | 91.17 | 66.17 | 154.45 | 109.63 | 50.01 |
| 112 | 27.78 | 18.33 | 57.63 | 350.18 | 307.44 | 276.26 | 92.07 | 94.04 | 79.14 | 205.14 | 129.1 | 39.15 |
| 113 | 92.90 | 87.33 | 30.88 | 341.91 | 289.66 | 226.97 | 72.83 | 69.85 | 86.39 | 138.86 | 99.43 | 41.73 |
| 114 | 63.78 | 94.33 | 101.78 | 222.98 | 259.66 | 222.63 | 71.40 | 63.67 | 54.28 | 160.19 | 128.38 | 45.23 |
| 115 | 87.56 | 53.33 | 138.77 | 272.66 | 145.00 | 279.70 | 67.89 | 63.22 | 50.39 | 102.71 | 140.29 | 46.56 |
| 116 | 27.22 | 52.13 | 80.24 | 276.52 | 258.13 | 390.04 | 90.16 | 79.80 | 79.43 | 141.80 | 106.75 | 36.62 |
| **RILs** | **UFG** | | | **TGN** | | | **SF (%)** | | | **YLD (gm)** | | |
|  | **Delhi** | **Kar** | **ADT** | **Delhi** | **Kar** | **ADT** | **Delhi** | **Kar** | **ADT** | **Delhi** | **Kar** | **ADT** |
| 117 | 76.78 | 94.44 | 63.48 | 328.77 | 316.00 | 172.05 | 76.65 | 70.11 | 63.10 | 102.90 | 120.02 | 33.53 |
| 118 | 139.44 | 93.22 | 98.28 | 444.00 | 295.66 | 279.79 | 68.59 | 68.47 | 64.87 | 208.70 | 106.29 | 50.08 |
| 119 | 35.65 | 45.56 | 89.07 | 237.10 | 269.45 | 202.46 | 84.96 | 83.09 | 56.01 | 158.94 | 113.69 | 40.73 |
| 120 | 70.44 | 29.20 | 65.35 | 373.44 | 328.40 | 335.88 | 81.14 | 91.11 | 80.54 | 113.08 | 113.33 | 62.15 |
| 121 | 71.22 | 24.11 | 76.06 | 282.92 | 271.67 | 272.36 | 74.83 | 91.13 | 72.07 | 209.68 | 106.65 | 44.66 |
| 122 | 101.56 | 86.11 | 83.19 | 316.45 | 284.11 | 253.81 | 67.91 | 69.69 | 67.22 | 187.89 | 101.07 | 42.07 |
| 123 | 61.56 | 62.50 | 62.32 | 445.76 | 285.63 | 281.79 | 86.19 | 78.12 | 77.88 | 196.97 | 120.62 | 57.90 |
| 125 | 35.50 | 4.63 | 75.56 | 359.60 | 347.33 | 224.19 | 90.13 | 95.25 | 66.30 | 105.37 | 149.49 | 45.71 |
| 126 | 115.43 | 34.00 | 172.61 | 334.73 | 308.67 | 323.31 | 65.52 | 88.99 | 46.61 | 197.14 | 122.01 | 47.88 |
| 127 | 101.22 | 40.67 | 9.27 | 340.52 | 212.27 | 163.89 | 70.27 | 80.84 | 94.34 | 213.24 | 109.22 | 56.55 |
| 128 | 90.44 | 31.67 | 195.57 | 406.34 | 320.00 | 266.26 | 77.74 | 90.10 | 26.55 | 173.28 | 121.35 | 64.91 |
| 129 | 90.71 | 35.44 | 113.53 | 344.71 | 245.44 | 321.12 | 73.69 | 85.56 | 64.65 | 104.70 | 124.52 | 52.59 |
| 131 | 51.44 | 74.98 | 79.28 | 340.54 | 331.97 | 259.79 | 84.89 | 77.41 | 69.48 | 89.49 | 118.02 | 38.55 |
| 132 | 86.67 | 71.76 | 69.39 | 323.97 | 288.63 | 258.82 | 73.25 | 75.14 | 73.19 | 122.14 | 102.21 | 60.52 |
| 133 | 113.78 | 122.63 | 54.90 | 346.78 | 413.74 | 255.77 | 67.19 | 71.95 | 78.54 | 179.26 | 117.98 | 58.61 |
| 134 | 85.67 | 26.22 | 33.32 | 404.07 | 259.66 | 233.36 | 78.80 | 89.90 | 85.72 | 105.61 | 107.78 | 49.70 |
| 136 | 80.33 | 83.56 | 87.18 | 261.93 | 247.00 | 158.96 | 69.33 | 66.17 | 45.16 | 209.30 | 129.81 | 59.86 |
| 137 | 58.60 | 38.56 | 109.58 | 373.90 | 298.23 | 209.91 | 84.33 | 87.07 | 47.80 | 163.29 | 107.75 | 47.43 |
| 139 | 94.25 | 75.62 | 167.29 | 303.87 | 270.60 | 323.07 | 68.98 | 72.05 | 48.22 | 99.03 | 103.69 | 42.01 |
| 140 | 29.67 | 19.33 | 77.63 | 245.97 | 200.66 | 270.67 | 87.94 | 90.37 | 71.32 | 144.79 | 128.1 | 40.58 |
| 141 | 37.89 | 23.00 | 88.71 | 271.29 | 253.00 | 219.24 | 86.03 | 90.91 | 59.54 | 119.96 | 121.38 | 47.97 |
| 142 | 120.29 | 19.57 | 66.05 | 257.99 | 226.00 | 173.20 | 53.37 | 91.34 | 61.86 | 129.83 | 140.98 | 36.31 |
| 143 | 89.00 | 25.67 | 150.20 | 265.87 | 188.23 | 388.64 | 66.52 | 86.36 | 61.35 | 185.67 | 146.4 | 37.89 |
| 144 | 71.46 | 59.00 | 142.37 | 383.21 | 348.63 | 210.34 | 81.35 | 83.08 | 32.31 | 191.74 | 118.2 | 45.07 |
| 145 | 78.78 | 64.14 | 95.64 | 233.78 | 207.28 | 316.64 | 66.30 | 69.06 | 69.80 | 84.16 | 102.57 | 57.07 |
| 146 | 67.00 | 36.89 | 42.37 | 248.90 | 284.56 | 215.71 | 73.08 | 87.04 | 80.36 | 90.79 | 120.04 | 69.12 |
| 147 | 38.00 | 140.68 | 63.91 | 326.94 | 298.06 | 314.08 | 88.38 | 55.51 | 79.65 | 102.69 | 155.74 | 68.36 |
| 148 | 65.13 | 49.25 | 135.59 | 304.83 | 379.63 | 276.45 | 78.63 | 87.03 | 50.95 | 89.87 | 116.31 | 38.90 |
| 149 | 64.89 | 61.00 | 96.68 | 351.17 | 374.50 | 284.52 | 81.52 | 83.71 | 66.02 | 89.74 | 114.65 | 48.91 |
| **RILs** | **UFG** | | | **TGN** | | | **SF (%)** | | | **YLD (gm)** | | |
|  | **Delhi** | **Kar** | **ADT** | **Delhi** | **Kar** | **ADT** | **Delhi** | **Kar** | **ADT** | **Delhi** | **Kar** | **ADT** |
| 150 | 117.00 | 89.22 | 92.85 | 337.40 | 320.55 | 353.32 | 65.32 | 72.17 | 73.72 | 130.40 | 101.78 | 43.57 |
| 151 | 91.33 | 78.71 | 123.27 | 300.73 | 207.14 | 278.36 | 69.63 | 62.00 | 55.72 | 209.74 | 113.88 | 45.68 |
| 152 | 84.10 | 62.44 | 98.99 | 383.20 | 210.44 | 231.83 | 78.05 | 70.33 | 57.30 | 105.21 | 135.79 | 47.43 |
| 153 | 157.56 | 77.34 | 138.68 | 379.46 | 279.21 | 291.46 | 58.48 | 72.30 | 52.42 | 100.67 | 120.36 | 41.80 |
| 154 | 90.89 | 109.89 | 150.71 | 359.39 | 221.89 | 330.93 | 74.71 | 50.48 | 54.46 | 95.90 | 117.79 | 55.42 |
| 155 | 80.22 | 76.00 | 106.28 | 369.24 | 298.00 | 225.89 | 78.27 | 74.50 | 52.95 | 133.11 | 103.32 | 57.93 |
| 156 | 58.56 | 70.89 | 86.22 | 374.36 | 195.56 | 246.70 | 84.36 | 63.75 | 65.05 | 106.83 | 97.08 | 33.57 |
| 157 | 93.89 | 49.60 | 112.90 | 378.98 | 279.30 | 250.53 | 75.23 | 82.24 | 54.94 | 128.31 | 105.1 | 67.93 |
| 158 | 73.25 | 18.44 | 68.92 | 382.23 | 206.00 | 226.39 | 80.84 | 91.05 | 69.56 | 100.55 | 127.43 | 42.98 |
| 159 | 36.44 | 28.86 | 39.17 | 366.34 | 180.43 | 363.76 | 90.05 | 84.00 | 89.23 | 136.20 | 112.42 | 63.90 |
| 160 | 163.73 | 45.89 | 196.78 | 538.31 | 327.11 | 317.42 | 65.20 | 85.97 | 38.01 | 157.36 | 108.89 | 64.32 |
| 161 | 50.56 | 61.79 | 76.94 | 260.26 | 283.25 | 164.33 | 80.57 | 78.19 | 53.18 | 80.43 | 108.5 | 30.05 |
| 162 | 68.50 | 75.62 | 121.23 | 216.59 | 252.75 | 353.21 | 68.37 | 70.08 | 65.68 | 197.97 | 123.25 | 34.97 |
| 163 | 42.56 | 35.86 | 132.34 | 382.26 | 357.58 | 230.80 | 88.87 | 89.97 | 42.66 | 116.36 | 106.17 | 44.38 |
| 164 | 65.40 | 73.00 | 120.39 | 386.07 | 288.11 | 397.99 | 83.06 | 74.66 | 69.75 | 114.45 | 111.63 | 35.98 |
| 165 | 103.67 | 39.00 | 150.03 | 303.23 | 236.00 | 291.68 | 65.81 | 83.47 | 48.56 | 125.56 | 120.57 | 43.20 |
| 166 | 86.67 | 65.56 | 101.91 | 339.05 | 297.08 | 268.14 | 74.44 | 77.93 | 61.99 | 189.72 | 121.71 | 63.51 |
| 167 | 152.82 | 97.62 | 153.45 | 374.28 | 299.04 | 294.97 | 59.17 | 67.36 | 47.98 | 150.36 | 122.91 | 49.91 |
| 168 | 43.50 | 53.67 | 160.35 | 200.18 | 266.23 | 270.44 | 78.27 | 79.84 | 40.71 | 114.27 | 104.9 | 40.86 |
| 169 | 129.44 | 54.00 | 22.73 | 356.75 | 247.33 | 114.21 | 63.72 | 78.17 | 80.84 | 113.36 | 120.61 | 36.64 |
| 170 | 153.63 | 29.67 | 82.87 | 303.03 | 358.23 | 268.59 | 42.84 | 91.72 | 69.15 | 84.15 | 128.54 | 38.92 |
| 171 | 153.44 | 45.88 | 195.26 | 416.68 | 279.65 | 447.78 | 63.18 | 83.59 | 55.68 | 131.93 | 127.46 | 49.25 |
| 172 | 94.33 | 65.61 | 35.86 | 304.53 | 287.37 | 192.93 | 69.02 | 77.17 | 81.41 | 90.53 | 127.89 | 42.72 |
| 173 | 102.67 | 35.75 | 147.44 | 330.57 | 260.13 | 310.75 | 68.94 | 86.26 | 52.55 | 142.16 | 124.75 | 47.26 |
| 174 | 138.22 | 116.89 | 49.93 | 343.32 | 338.45 | 190.14 | 59.74 | 65.46 | 73.74 | 164.38 | 117.18 | 53.85 |
| 175 | 79.89 | 26.44 | 105.67 | 350.49 | 325.55 | 311.43 | 77.21 | 91.88 | 66.07 | 91.39 | 128.26 | 43.80 |
| 176 | 166.63 | 111.84 | 69.88 | 353.03 | 313.60 | 173.40 | 52.80 | 64.34 | 59.70 | 108.44 | 103.59 | 51.60 |
| 177 | 81.54 | 75.22 | 130.18 | 352.89 | 364.55 | 203.58 | 76.89 | 79.37 | 36.05 | 151.32 | 111.71 | 56.18 |
| 178 | 29.56 | 21.22 | 23.42 | 284.66 | 264.33 | 200.71 | 89.62 | 91.97 | 88.33 | 133.14 | 101.37 | 49.87 |
| **RILs** | **UFG** | | | **TGN** | | | **SF (%)** | | | **YLD (gm)** | | |
|  | **Delhi** | **Kar** | **ADT** | **Delhi** | **Kar** | **ADT** | **Delhi** | **Kar** | **ADT** | **Delhi** | **Kar** | **ADT** |
| 179 | 70.00 | 56.89 | 109.07 | 371.10 | 290.78 | 251.35 | 81.14 | 80.44 | 56.61 | 209.70 | 109.69 | 46.20 |
| 180 | 140.22 | 103.22 | 150.79 | 302.60 | 285.00 | 241.30 | 53.66 | 63.78 | 37.51 | 111.24 | 130.37 | 58.45 |

**Supplementary table 4: Principal components for grain number and yield attributing traits among RILs.**

| Statistics | | PC1 | PC2 | PC3 | PC4 | PC5 | PC6 | PC7 | PC8 | PC9 |
| --- | --- | --- | --- | --- | --- | --- | --- | --- | --- | --- |
| Standard deviation | | 1.73 | 1.38 | 1.21 | 1.06 | 0.78 | 0.72 | 0.59 | 0.21 | 0.02 |
| Proportion of Variance | | 0.33 | 0.21 | 0.16 | 0.13 | 0.07 | 0.06 | 0.04 | 0.00 | 0.00 |
| Cumulative Proportion | | 0.33 | 0.54 | 0.71 | 0.83 | 0.90 | 0.96 | 1.00 | 1.00 | 1.00 |
| Trait contribution | PH | 0.35 | 0.29 | 0.10 | -0.11 | -0.81 | -0.24 | 0.23 | -0.01 | 0.00 |
|  | PL | 0.14 | -0.16 | -0.56 | -0.45 | 0.09 | -0.58 | -0.30 | -0.06 | 0.00 |
|  | TN | 0.09 | 0.00 | 0.25 | -0.83 | 0.21 | 0.26 | 0.36 | 0.03 | 0.00 |
|  | PBN | 0.43 | 0.16 | 0.30 | -0.12 | -0.01 | 0.27 | -0.78 | 0.04 | -0.01 |
|  | FGN | 0.46 | 0.33 | -0.11 | 0.18 | 0.36 | -0.04 | 0.22 | -0.37 | 0.57 |
|  | UFG | 0.36 | -0.55 | 0.06 | 0.12 | -0.02 | -0.03 | 0.10 | 0.63 | 0.38 |
|  | TGN | 0.54 | -0.03 | -0.05 | 0.20 | 0.27 | -0.05 | 0.23 | 0.05 | -0.73 |
|  | SF | -0.13 | 0.67 | -0.20 | -0.02 | 0.18 | -0.06 | 0.01 | 0.68 | 0.01 |
|  | YLD | 0.11 | -0.05 | -0.69 | -0.04 | -0.24 | 0.67 | 0.02 | -0.02 | 0.00 |

PC, Principal component; FGN, Filled grain number; PBN, Primary branches number; PH, Plant height; PL, Panicle length; SF, Spikelet fertility; TGN, Total grain number; TN, Tiller number; UFG, Unfilled grain; YLD, Yield.

**Supplementary table 5: List of polymorphic markers used for genotyping the RILs**

| **Marker** | **Chromosome** | **Position (Mb)** | **Forward primer (5'-3')** | **Reverse primer (5'-3')** |
| --- | --- | --- | --- | --- |
| RM10217 | 1 | 3.96 | GCACTCACTCTCACTGATCACTTGC | CTCGTGTTGTGTTGGTGTGTTGC |
| RM8094 | 1 | 11.23 | AAGTTTGTACACATCGTATACA | CGCGACCAGTACTACTACTA |
| RM493 | 1 | 12.28 | TAGCTCCAACAGGATCGACC | GTACGTAAACGCGGAAGGTG |
| RM10843 | 1 | 13.77 | CACCTCTTCTGCCTCCTATCATGC | GTTTCTTCGCGAAATCGTGTGG |
| RM11060 | 1 | 19.2 | AAGCTTGCGTAGCGTAGGTGGTAGG | CCGGAATATTCTCACCGACCTTGC |
| RM9 | 1 | 23.32 | GGTGCCATTGTCGTCCTC | ACGGCCCTCATCACCTTC |
| RM5 | 1 | 23.97 | TGCAACTTCTAGCTGCTCGA | GCATCCGATCTTGATGGG |
| RM12230 | 1 | 42.38 | CACCTGTTGTGAATCTGTGATCG | CGGTGGAGCTTGTTATACTACTATGG |
| RM3732 | 2 | 4.4 | ATCCACAAACTCAGATGGGC | TGCCACGCGATTGAAGAC |
| RM12705 | 2 | 6.26 | ACCGCGTCAGCTATAGTTTCATCG | GCATGCTGGAGGGCTACATGG |
| RM424 | 2 | 11.38 | TTTGTGGCTCACCAGTTGAG | TGGCGCATTCATGTCATC |
| RM13254 | 2 | 17.86 | CAGAAGCCTTCATCCCAGATAGG | CAAACGGGATCACCATCTTCC |
| RM2634 | 2 | 20.49 | GATTGAAAATTAGAGTTTGCAC | TGCCGAGATTTAGTCAACTA |
| HvSSR02-59 | 2 | 22.14 | GGTTGAATGTGGCATTTACT | TAGATGGTAAACCTGCCACT |
| RM13672 | 2 | 26.02 | CAGCAGCAGCATCTCCGAGTACC | ATCAGTGGCACCAACCTGACACG |
| RM6 | 2 | 29.57 | GTCCCCTCCACCCAATTC | TCGTCTACTGTTGGCTGCAC |
| RM5916 | 2 | 34.05 | GCTATAAGAATCGTATTAAG | TACTGCTATTAAAGTCAGAA |
| RM207 | 2 | 35.36 | CCATTCGTGAGAAGATCTGA | CACCTCATCCTCGTAACGCC |
| RM489 | 3 | 4.33 | ACTTGAGACGATCGGACACC | TCACCCATGGATGTTGTCAG |
| RM3716 | 3 | 6.83 | GTCGTTCGGTTGACTCGTTG | CACACATATATACCCCCCCC |
| OSR13 | 3 | 7.12 | CATTTGTGCGTCACGGAGTA | AGCCACAGCGCCCATCTCTC |
| RM7 | 3 | 9.28 | TTCGCCATGAAGTCTCTCG | CCTCCCATCATTTCGTTGTT |
| RM3646 | 3 | 21.99 | ACTAGAGCACCCTCGCTGAG | CTCAGCCACCCCATCAAC |
| RM168 | 3 | 28.09 | TGCTGCTTGCCTGCTTCCTTT | GAAACGAATCAATCCACGGC |
| HvSSR.3-82 | 3 | 30.34 | GCAAACGACACAAGTCATTA | ATAGTGCCCTTTCTTTCACA |
| RM520 | 3 | 30.91 | AGGAGCAAGAAAAGTTCCCC | GCCAATGTGTGACGCAATAG |
| **Marker** | **Chromosome** | **Position (Mb)** | **Forward primer (5'-3')** | **Reverse primer (5'-3')** |
| HvSSR03-86 | 3 | 31.94 | GCAGTCTTCCGAATTGATAC | TCAATGATTTATTATGGCCC |
| RM1230 | 3 | 32.75 | GGGTGGTGTGAGCTTTTCTC | TTCCACTTCGACAACCCTTC |
| RM335 | 4 | 0.68 | GTACACACCCACATCGAGAAG | GCTCTATGCGAGTATCCATGG |
| RM16302 | 4 | 1 | CCGATCTGGAAACAGCCACAGC | CGCAAGCCAAGTCGCAATCC |
| HvSSR04-19 | 4 | 6.56 | TTGGAATCCTTGAGAAGAAA | TCAAATACGATGACGTACCA |
| RM16775 | 4 | 17.57 | TTCTAAGCCGCTGTGAATAACC | GGTAACCAATGAGGCTGATACC |
| nksssr04-11 | 4 | 30.68 | CCATCAGTTGAAGGGCTCTC | CTTTTATGGCATGGGCAACT |
| RM5709 | 4 | 31.87 | CTGAATTTATTATAGGACGGAAG | CATAGTATTGGATTGGACACG |
| RM1153 | 4 | 32.84 | ACCAACGCCAAAAGCTACTG | TACTCGCCCTGCATGAGC |
| RM127 | 4 | 34.52 | GTGGGATAGCTGCGTCGCGTCG | AGGCCAGGGTGTTGGCATGCTG |
| RM567 | 4 | 34.53 | CTCTTCACTCACTCACCATGG | CTCTTCACTCACTCACCATGG |
| RM280 | 4 | 34.98 | ACACGATCCACTTTGCGC | TGTGTCTTGAGCAGCCAGG |
| RM122 | 5 | 0.31 | GAGTCGATGTAATGTCATCAGTGC | GAAGGAGGTATCGCTTTGTTGGAC |
| RM17819 | 5 | 1.61 | TTGCCTCATGTTTGCTTCATCC | AGCTGACAACGACGACACTGC |
| HvSSR05-12 | 5 | 3.16 | TCCTCTACAGTTGTCTGCCT | CATTCCTCTCCACTTTCTTG |
| HvSSR05-21 | 5 | 6.8 | GGATCACCAGAGATGAAGAA | AAACTACTCCAATCTGCCAA |
| RM18175 | 5 | 8.77 | AAAGTCACTCACACGCCGTACTCC | ACGAATGAGTCCGACCATGACC |
| HvSSR05-30 | 5 | 13.46 | TACGACGGACGATTAAAGTT | GCTAACTCATTCATCTCGCT |
| RM146 | 5 | 18.05 | CTATTATTCCCTAACCCCCATACCCTCC | AGAGCCACTGCCTGCAAGGCCC |
| RM164 | 5 | 19.19 | TCTTGCCCGTCACTGCAGATATCC | GCAGCCCTAATGCTACAATTCTTC |
| RM3663 | 5 | 21.36 | CATCAACCTCCACGAACATG | CTCGGTGGTGATCCTCCTC |
| RM3476 | 5 | 23.84 | GATTCTCGTCGTAATCAAGA | ATCCACGGTTAAGATAAATG |
| RM26 | 5 | 27.34 | GAGTCGACGAGCGGCAGA | CTGCGAGCGACGGTAACA |
| RM31 | 5 | 28.61 | GATCACGATCCACTGGAGCT | AAGTCCATTACTCTCCTCCC |
| RM19274 | 6 | 1.01 | CCTGTGAATGACAACCCATGC | GTATGAGCCAGATTAGCGGTTGC |
| RM190 | 6 | 1.76 | CTCTCTCACCATTCCTTCAG | GATCTGAATAAGAGGGGAAAC |
| RM204 | 6 | 3.16 | GTGACTGACTTGGTCATAGGG | GCTAGCCATGCTCTCGTACC |
| RGNMS2221 | 6 | 5.23 | AATTTACTGAAATTTCGATTGG | ATAATTAATCACATCTGCCCAC |
| RM7311 | 6 | 11.04 | AGTGGTCGTTGAACTCGGAG | TCGTGGCGCCTTTAATCTC |
| **Marker** | **Chromosome** | **Position (Mb)** | **Forward primer (5'-3')** | **Reverse primer (5'-3')** |
| RM193 | 6 | 18.08 | CGCCTCTTCTTCCTCGCCTCCG | CGGGTCCATCCCCCCTCTCCTC |
| RM528 | 6 | 26.55 | GGCATCCAATTTTACCCCTC | AAATGGAGCATGGAGGTCAC |
| HvSSR07-07 | 7 | 1.55 | GAACCGTAGGTTACAGTGGA | AGAGCTTGATTCAATCGGTA |
| RM5344 | 7 | 1.9 | ACGAACGGGAGCAAGGTC | CTCTCAACCAAGACGCCTTC |
| RM2 | 7 | 16.02 | ACGTGTCACCGCTTCCT | ATGTCCGGGATCTCATCG |
| RM432 | 7 | 18.95 | TTCTGTCTCACGCTGGATTG | AGCTGCGTACGTGATGAATG |
| RM10 | 7 | 22.19 | TTGTCAAGAGGAGGCATCG | CAGAATGGGAAATGGGTCC |
| RM1235 | 8 | 1.2 | AGCAGAGGAGGAGATGATGG | GGACCAAAACGAAGCTATCC |
| RM25 | 8 | 4.38 | GGAAAGAATGATCTTTTCATGG | CTACCATCAAAACCAATGTTC |
| RM22837 | 8 | 12.37 | ACCTGGGTCAGATGTCTGTTTGG | GGTAGAGCTCCATCCATCTTAGTGC |
| RM210 | 8 | 22.47 | TCACATTCGGTGGCATTG | CGAGGATGGTTGTTCACTTG |
| RM80 | 8 | 24.47 | TTGAAGGCGCTGAAGGAG | CATCAACCTCGTCTTCACCG |
| RM23449 | 8 | 25.69 | CCAATGGTACGTGTGTTCATCC | CGATCGGAATCTCATCGAAGG |
| RM264 | 8 | 27.92 | GTTGCGTCCTACTGCTACTTC | GATCCGTGTCGATGATTAGC |
| RM444 | 9 | 5.92 | GCTCCACCTGCTTAAGCATC | TGAAGACCATGTTCTGCAGG |
| RM6920 | 9 | 7.00 | AGAGCGTACCACAAATGAGG | AATCGTATTGCCAGCGAGAC |
| RM3700 | 9 | 15.42 | AAATGCCCCATGCACAAC | TTGTCAGATTGTCACCAGGG |
| RM242 | 9 | 18.81 | GGCCAACGTGTGTATGTCTC | TATATGCCAAGACGGATGGG |
| RM201 | 9 | 20.17 | CTCGTTTATTACCTACAGTACC | CTACCTCCTTTCTAGACCGATA |
| RM215 | 9 | 21.18 | CAAAATGGAGCAGCAAGAGC | TGAGCACCTCCTTCTCTGTAG |
| RM216 | 10 | 5.35 | GCATGGCCGATGGTAAAG | TGTATAAAACCACACGGCCA |
| RM5348 | 10 | 8.48 | AATCCGATAGGAGTACCGCC | AAGTGTATGGGCTGGAATGG |
| RGNMS3249 | 10 | 10.23 | TGGGTTCTTATATGGATTCTTC | AATAGCTGAAATTCGGAATAAA |
| RM147 | 10 | 20.94 | TACGGCTTCGGCGGCTGATTCC | CCCCCGAATCCCATCGAAACCC |
| RM200 | 10 | 12.38 | CGCTAGGGAATTTGGATTGA | CGATGAGCAGGTATCGATGAGAAG |
| RM286 | 11 | 0.38 | GGCTTCATCTTTGGCGAC | CCGGATTCACGAGATAAACTC |
| RM1812 | 11 | 2.41 | CAGCTAGTGAGCTCCTAGTG | GCTAACCCACCAACTTATTC |
| RM332 | 11 | 2.82 | GCGAAGGCGAAGGTGAAG | CATGAGTGATCTCACTCACCC |
| HvSSR11-13 | 11 | 5.91 | TGAAACCACAATGAGTCAAA | GCCCTAAACCCAAATAGAAG |
| **Marker** | **Chromosome** | **Position (Mb)** | **Forward primer (5'-3')** | **Reverse primer (5'-3')** |
| HvSSR11-25 | 11 | 6.04 | TCGAAGTCCAAATTTCCTTA | GATTGGAGTTTGAACGATGT |
| RM26499 | 11 | 11 | TCTCCTTTATCCGTGACCTGAGC | ATGATGTGTCTTGCCACATAGACG |
| RM5590 | 11 | 11.68 | TGGATAAGCGATTGAGGTAG | CGTTATAATGAGGGAGGGAG |
| RM287 | 11 | 16.76 | TTCCCTGTTAAGAGAGAAATC | GTGTATTTGGTGAAAGCAAC |
| RM21 | 11 | 19.63 | ACAGTATTCCGTAGGCACGG | GCTCCATGAGGGTGGTAGAG |
| RGNMS3232 | 11 | 19.76 | ACTGGCTGTACCGTACTAGTTT | CTTTCTATCTTCGCTCTCAGTC |
| RM206 | 11 | 22.01 | CCCATGCGTTTAACTATTCT | CGTTCCATCGATCCGTATGG |
| RM6965 | 11 | 24.63 | TCATTTGGATCATAAGCTGG | TTGGATGAGATAACCAATGC |
| RM27150 | 11 | 24.71 | ATTCAGGCTCGCTTACCATCTCC | CCTCTGCTTGTCCCAAATCACC |
| RM144 | 11 | 28.28 | TGCCCTGGCGCAAATTTGATCC | GCTAGAGGAGATCAGATGGTAGTGCATG |
| RM101 | 12 | 8.82 | GTGAATGGTCAAGTGACTTAGGTGGC | ACACAACATGTTCCCTCCCATGC |
| RM1047 | 12 | 11.22 | ATTACAGAACCCCACTCCCC | CATCATCTTAGCCCCCAGTG |
| RM28032 | 12 | 13.64 | ACGACACGGATGAGTTCAGTGG | GGATCTGAGAGGAAGAGGGAAGG |
| RM217 | 12 | 17.91 | ATCGCAGCAATGCCTCGT | GGGTGTGAACAAAGACAC |
| RM28204 | 12 | 18.28 | CATTCTACCGATGATTGCAGAGG | CTACATTAAGCGTGAGCGACAGC |
| RM1246 | 12 | 19.08 | CTCGATCCCCTAGCTCTC | TCACCTCGTTCTCGATCC |
| RM6217 | 12 | 22.63 | GCAGCAAGAGCAAGAAATCC | GTTCCTGCCGTACCAGCAG |
| RM1103 | 12 | 23.53 | CAGCTGCTGCTACTACACCG | CTACTCCACGTCCATGCATG |
| RM1226 | 12 | 27.31 | TCCCTCACCCTCACTCTCAC | TCTTGTTGCTTGTGCTGTCC |

Mb, Mega base pair

**Supplementary table 6: Chromosome-wise number of the markers used for construction of linkage map, map length, and marker density.**

| Chromosome | Markers used for polymorphism survey | Number of markers amplified | Polymorphic Markers | Length (in cM) | Marker density (cM) |
| --- | --- | --- | --- | --- | --- |
| 1 | 125 | 105 | 8 | 177.95 | 22.24 |
| 2 | 119 | 93 | 10 | 215.34 | 21.53 |
| 3 | 117 | 89 | 10 | 212.66 | 21.27 |
| 4 | 106 | 99 | 10 | 229.76 | 22.98 |
| 5 | 115 | 101 | 12 | 222.59 | 18.55 |
| 6 | 106 | 78 | 7 | 230.41 | 32.92 |
| 7 | 108 | 81 | 5 | 63.75 | 12.75 |
| 8 | 118 | 87 | 7 | 224.01 | 32.00 |
| 9 | 126 | 113 | 6 | 234.57 | 39.10 |
| 10 | 118 | 77 | 5 | 219.01 | 43.80 |
| 11 | 135 | 93 | 14 | 222.59 | 15.90 |
| 12 | 132 | 67 | 9 | 162.43 | 18.05 |
| Total | 1425 | 1083 | 103 | 2415.07 |  |

cM, Centi morgan.
